# A single region of the *Phytophthora infestans* avirulence effector Avr3b functions in both cell death induction and plant immunity suppression

**DOI:** 10.1111/mpp.13298

**Published:** 2023-01-25

**Authors:** Biao Gu, Wenxin Gao, Zeqi Liu, Guangda Shao, Qin Peng, Yinyu Mu, Qinhu Wang, Hua Zhao, Jianqiang Miao, Xili Liu

**Affiliations:** ^1^ State Key Laboratory of Crop Stress Biology for Arid Areas and College of Plant Protection Northwest A&F University Yangling China

**Keywords:** cell death, effector‐triggered immunity (ETI), hypersensitive response (HR), PAMP‐triggered immunity (PTI), *Phytophthora infestans*, RxLR effector

## Abstract

As a destructive plant pathogen, *Phytophthora infestans* secretes diverse host‐entering RxLR effectors to facilitate infection. One critical RxLR effector, PiAvr3b, not only induces effector‐triggered immunity (ETI), which is associated with the potato resistance protein StR3b, but also suppresses pathogen‐associated molecular pattern (PAMP)‐triggered immunity (PTI). To date, the molecular basis underlying such dual activities remains unknown. Based on phylogenetic analysis of global *P. infestans* isolates, we found two PiAvr3b isoforms that differ by three amino acids. Despite this sequence variation, the two isoforms retain the same properties in activating the StR3b‐mediated hypersensitive response (HR) and inhibiting necrosis induced by three PAMPs (PiNpp, PiINF1, and PsXeg1) and an RxLR effector (Pi10232). Using a combined mutagenesis approach, we found that the dual activities of PiAvr3b were tightly linked and determined by 88 amino acids at the C‐terminus. We further determined that either the W60 or the E134 residue of PiAvr3b was essential for triggering StR3b‐associated HR and inhibiting PiNpp‐ and Pi10232‐associated necrosis, while the S99 residue partially contributed to PTI suppression. Additionally, nuclear localization of PiAvr3b was required to stimulate HR and suppress PTI, but not to inhibit Pi10232‐associated cell death. Our study revealed that PiAvr3b suppresses the plant immune response at different subcellular locations and provides an example in which a single amino acid of an RxLR effector links ETI induction and cell death suppression.

## Graphical abstract

*Phytophthora infestans* RxLR effector Avr3b activates effector‐triggered immunity and suppresses pathogen‐associated molecular pattern‐triggered immunity in the nucleus, while nuclear localization of Avr3b is not required for inhibition of effector‐induced cell death.

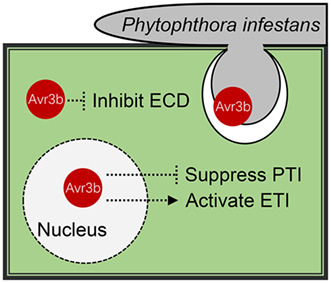

## 1 INTRODUCTION

The oomycete pathogen *Phytophthora infestans* causes late blight diseases and has threatened global food production since the 19th century Irish Potato Famine. As the most destructive pathogen of potatoes, *P. infestans* is infamous for its rapid spread and frequent resistance (Fry, 2008; Fry et al., 2015). *P. infestans* manipulates the host immune system by orchestrating an arsenal of cell‐entering RxLR effectors. Some RxLR effectors are avirulence (Avr) proteins that exhibit specific gene‐for‐gene interactions with their cognate host resistance (R) proteins, leading to effector‐triggered immunity (ETI) and often eliciting a hypersensitive response (HR) (Naveed et al., 2020). Most characterized R proteins in the *Phytophthora*–plant pathosystem are nucleotide‐binding, leucine‐rich‐repeat receptors (NLRs) that indirectly interact with corresponding Avr proteins. For example, interactions between PiAvr2 and R2 and between PiAvrvnt1 and Rpivnt1 are indirect and mediated by BSL1 and GLYK, respectively (Gao et al., 2020; Saunders et al., 2012). Multiple components of the ETI signalling pathway, such as BAK1, HSP90, NPK, RAR1, SGT1, MEK2, WIPK, and NDR1, are required for RxLR effector‐associated hypersensitivity (Oh et al., 2014; Situ et al., 2020; Yang et al., 2017, 2021). Notably, *Nicotiana benthamiana* SGT1 is indispensable for HR associated with several *R* genes (such as *R1*, *R2*, *R3a*, *RB*, *Rpiblb2*, and *Rpivnt1*) in response to the cognate Avr proteins (Wu et al., 2017). Meanwhile, pathogen‐associated molecular pattern (PAMP)‐induced cell death, triggered by PAMPs such as PiNpp and INF1, relies on *SGT1* and *HSP90* in *N. benthamiana* (Kanneganti et al., 2006). However, the exact pathways regulating effector‐ and PAMP‐associated cell death remain obscure.

Sequence polymorphisms, gene deletion, copy number variation, and transcriptional dynamics of *AVR* genes result in a spectrum of virulence among *Phytophthora* isolates (Dong & Ma, 2021). For instance, the residues at positions 80 and 103 of Avr3a vary between avirulent and virulent isolates of *P. infestans* and determine elicitation of the R protein R3a (Armstrong et al., 2005). Deletion or silencing of *PiAVR2* results in loss of avirulence in *P. infestans* on plants harbouring the *R2* gene (Gilroy et al., 2011). In several studies, the dual activities of RxLR effectors related to cell death activation and host immune suppression were linked to different amino acids. For example, the K80 and I103 residues of *P. infestans* PiAvr3a^KI^ determined R3a‐mediated HR, while the Y147 residue was required for PiINF1 suppression but not R3a activation (Bos et al., 2009). Mutational analysis of *Phytophthora sojae* Avh238 indicated that the 79th residue determined cell death‐inducing activity, and the 53rd amino acid in its C‐terminal region was critical for promoting infection (Yang et al., 2017).

Studies have also shown that subcellular localization of effectors impacts their activity. Transient expression of 52 infection‐inducing RxLR effectors in *N. benthamiana* revealed that most effectors localize in the cytoplasm, nuclei, or associate with the plasma membrane, while only a few localize in other subcellular compartments (Wang et al., 2019). The diversity of localization patterns suggests that subcellular localization plays a critical role in plant defence responses. On the other hand, R protein recognition also promotes the relocalization of the corresponding effectors. In *N. benthamiana*, the nuclear‐cytosolic RxLR effector PiAvr3a^KI^ moved to endosomes with R3a to initiate HR (Engelhardt et al., 2012). Activating R1‐mediated HR required nuclear localization of Avr1, while Avr1‐mediated cell death suppression required its localization in the cytoplasm (Du et al., 2015). Similarly, nuclear localization of *P. sojae* Avh238 was essential for triggering cell death, while cytoplasmic localization of the effector was needed for Avh238‐mediated suppression of INF1‐triggered cell death (Yang et al., 2017).

*P. infestans PiAvr3b* encodes a cytoplasmic RxLR effector and activates multiple *R* genes that have been extensively employed in the disease‐resistance breeding of potatoes (Rietman, 2011). Nevertheless, PiAvr3b elicits hypersensitive cell death in wild potato (*Solanum pinnatisectum*), which is highly resistant to late blight disease (Gu et al., 2020), suggesting cognate *R* genes are valuable for durable resistance. The cloning of *StR3b* and its matching avirulence gene *PiAVR3b* (Li et al., 2011) provides a new tool for understanding the coevolution of host and pathogen machinery in potato and *P. infestans*, respectively. Without a corresponding StR3b protein, PiAvr3b could function as a virulence factor to suppress immune responses induced by the PAMP PiNpp (Zuluaga et al., 2016) and the RxLR effector Pi22798 (Wang et al., 2017), suggesting a potential virulence function of this protein. *PiAVR3b* deletions and single‐nucleotide polymorphisms (SNPs) (Rietman, 2011; Thilliez et al., 2019) may indicate adaptive changes occurring in the genomes of *P. infestans* isolates that may promote host immune surveillance evasion. However, the variation patterns and structural basis underlying the dual activities of PiAvr3b remain largely unknown.

In this study, we examined sequence variation and changes in the transcription of *PiAvr3b* and analysed the regions and residues responsible for ETI induction and PTI suppression. We also investigated the correlation between subcellular localization of PiAvr3b and its activities in regulating plant defence. Our study revealed that the C‐terminal region of PiAvr3b promotes infection by interfering with multiple subcellular processes of plant immunity, and correct subcellular localization is critical for the dual activities of PiAvr3b. Our findings provide an example in which a single amino acid in an RxLR effector regulates ETI elicitation and PTI suppression.

## 2 RESULTS

### 2.1 SNPs, genomic deletion, and transcriptional variation of *PiAVR3b* among *P. infestans* field isolates

**FIGURE 1:**
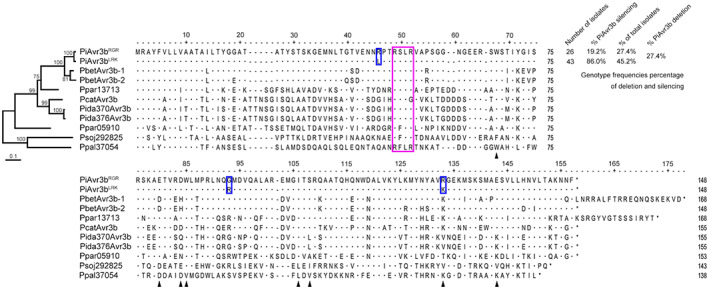
Sequence analysis of PiAvr3b isoforms and orthologs in selected *Phytophthora* species. The purple rectangle encompasses RxLR motifs, and the blue rectangles indicate the amino acids that differ between PiAvr3b^RGR^ and PiAvr3b^LRK^. Phylogenetic analysis of PiAvr3b and its homologs found in *Phytophthora*. The two PiAvr3b‐like effectors Psoj292825 and Ppal37054 from *P. sojae* and *P. palmivora*, respectively, were used as outgroups. Numbers in the phylogenetic tree indicate bootstrap values, and the scale bar represents 20% weighted sequence divergence. Numbers in the upper right corner indicate genotype frequencies of PiAvr3b alleles in different *P. infestans* isolates. Black arrows indicate residues subject to the following mutation analysis.

To investigate *PiAVR3b* sequence variation in *P. infestans*, we PCR‐amplified the *PiAVR3b* coding regions from 95 geographically distinct isolates (Table S1). We could not amplify the *AVR3b* open reading frames (ORFs) from 27 isolates, indicating that the loci may be disrupted in these isolates (Figure S1). Sanger sequencing of the remaining 68 PCR‐amplicons revealed that the amino acid polymorphisms occurred mainly at the 46th, 93rd, and 133rd residues, which classified PiAvr3b into two isoforms: PiAvr3b^RGR^ and PiAvr3b^LRK^ (Figure 1). We further examined the transcription of the *AVR3b* loci that were amplified and found that AVR3b was silenced in 42 isolates (Figure S2 and Table S2). Strikingly, we found that the isolates collected recently showed a much higher frequency of *PiAVR3b* disruption or silencing (Table S2), indicating a dominant lineage alternation in the Chinese *P. infestans* population.

Further BLAST analysis using the *AVR3b* ORF as the query sequence revealed a paralogous gene, *Pi18221*. Compared to *PiAVR3b*, *Pi18221* lacks two nucleotides, which causes a frameshift and is predicted to truncate the protein by 43 amino acids at the C‐terminus (Figure S2a,b). Interestingly, our transcriptional analysis indicated that *Pi18221* transcripts were present in both *PiAVR3b*‐silencing and *PiAVR3b*‐expressing isolates (Figure S3), suggesting that Pi18221 may be a functional effector protein.

To further understand the divergence of PiAvr3b, we conducted homolog searches against other *Phytophthora* species. PiAvr3b orthologs were identified in several closely related species, including *Phytophthora cactorum*, *Phytophthora idaei*, *Phytophthora parasitica*, *Phytophthora betacei*, and *Phytophthora mirabilis* (Figure 1 and Table S3). The evolution of PiAvr3b correlated with the current speciation model of these *Phytophthora* species. In addition, we found several PiAvr3b‐like effectors in other *Phytophthora* species, such as Psoj292825 in *P. sojae* and Ppal37054 in *Phytophthora palmivora*, through BLAST searches. All of the PiAvr3b‐like effectors identified showed low sequence similarity (identity <30%) to PiAvr3b (Figure 1). For these PiAvr3b‐like effector proteins, reciprocal homolog searches were unsuccessful, identifying only different effectors, Pi05910 and Pi19831, in *P. infestans* (Table S3). Thus, our results indicate that PiAvr3b is only conserved in the *Phytophthora* Clade I and displays two isoforms and transcriptional variation.

### 2.2 PiAvr3b orthologues trigger distinct cell deaths indirectly mediated by StR3b

**FIGURE 2:**
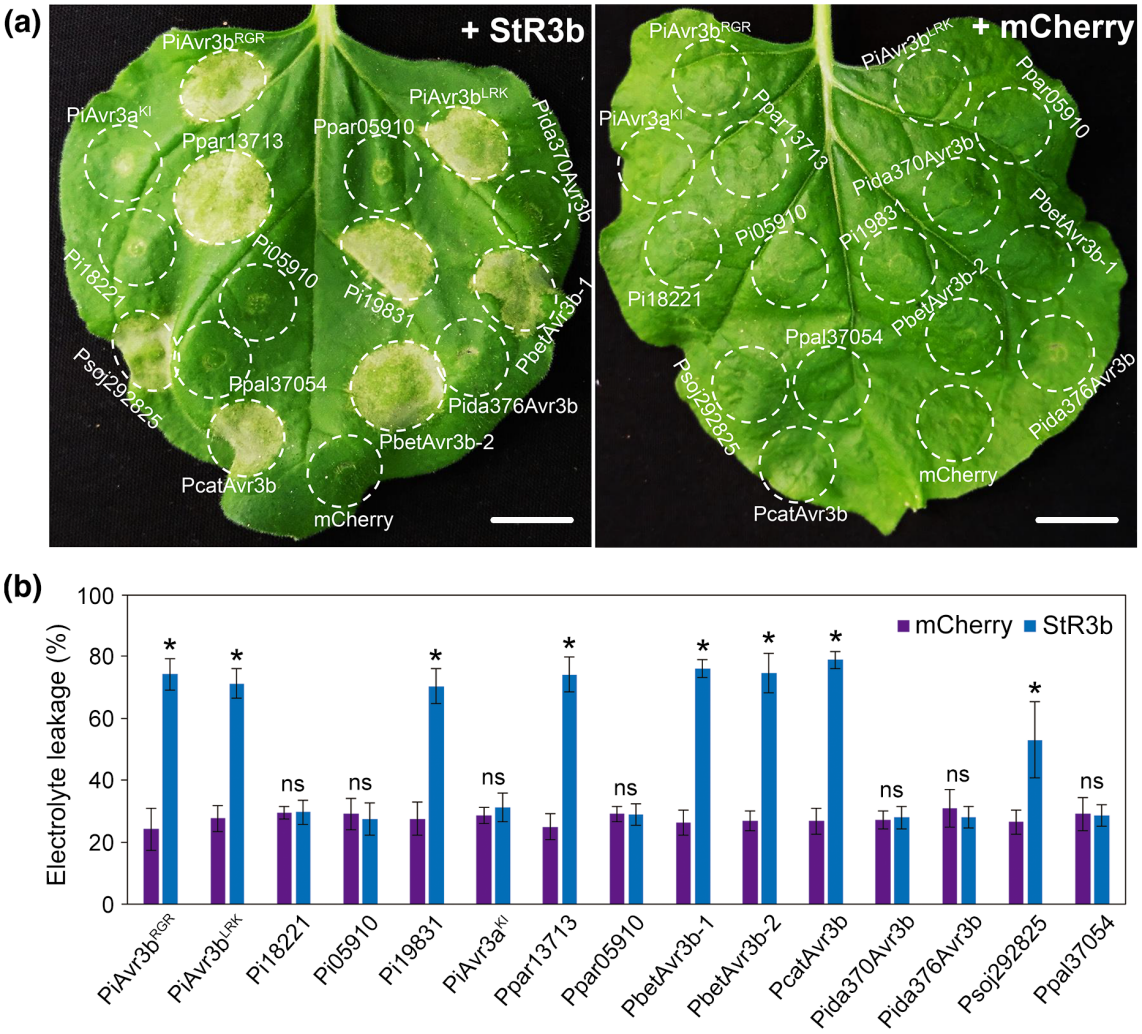
PiAvr3b intra‐ and interspecies homologs stimulate different StR3b‐mediated hypersensitive responses (HR). (a) Co‐expression of StR3b and mCherry‐tagged PiAvr3b homologs in *Nicotiana benthamiana*. PiAvr3a^KI^ and mCherry were used as controls. Images were taken 3 days postagroinfiltration (dpa). All experiments were performed in triplicate and showed similar results. Scale bars, 1 cm. (b) Electrolyte leakage from infiltration sites was measured as a percentage of leakage from boiled samples. Wilcoxon rank‐sum test was used for statistical analysis. Error bars represent *SD*; asterisks indicate 0.01 < *p* < 0.05. ns, not significant.

Previous studies demonstrated that PiAvr3b^RGR^ triggers StR3b‐mediated HR in *N. benthamiana* and wild potato cultivars (Gu et al., 2020; Li et al., 2011). To examine whether the polymorphisms identified in other PiAvr3b variants affect StR3b‐dependent HR in plants, we co‐expressed StR3b with mCherry‐tagged PiAvr3b variants in *N. benthamiana*. As shown in Figure 2, both PiAvr3b^RGR^ and PiAvr3b^LRK^ elicited substantial cell death, whereas the truncated PiAvr3b paralogue Pi18221 failed to stimulate StR3b‐HR. Most PiAvr3b orthologs from other *Phytophthora* species showed a necrotic phenotype similar to that of PiAvr3b, except for Ppar05910, Pida370Avr3b, and Pida376Avr3b (Figure 2a and Table S3). Interestingly, the two PiAvr3b‐like effectors, Psoj292825 (from *P. sojae*) and Pi19831 (from *P. infestans*), also triggered HR. *N. benthamiana* leaves expressing Pi05910, Ppar05910, Pida370Avr3b, or Pida376Avr3b showed significantly less ion leakage than other PiAvr3b orthologs (Figure 2b). Western blot analysis verified expression of each effector protein fusion (Figure S4), suggesting that the difference in StR3b‐dependent HR was not attributable to the instability of PiAvr3b orthologs or PiAvr3b‐like proteins.

To test whether PiAvr3b physically binds to StR3b, we conducted a yeast two‐hybrid assay and found that neither PiAvr3b^RGR^ nor PiAvr3b^LRK^ bound to StR3b (Figure S5). Our results indicate that StR3b possesses a broad effector recognition capacity and may interact with effector proteins indirectly.

### 2.3 HSP90 and SGT1 are essential for PiAvr3b‐triggered ETI in *N. benthamiana*

**FIGURE 3:**
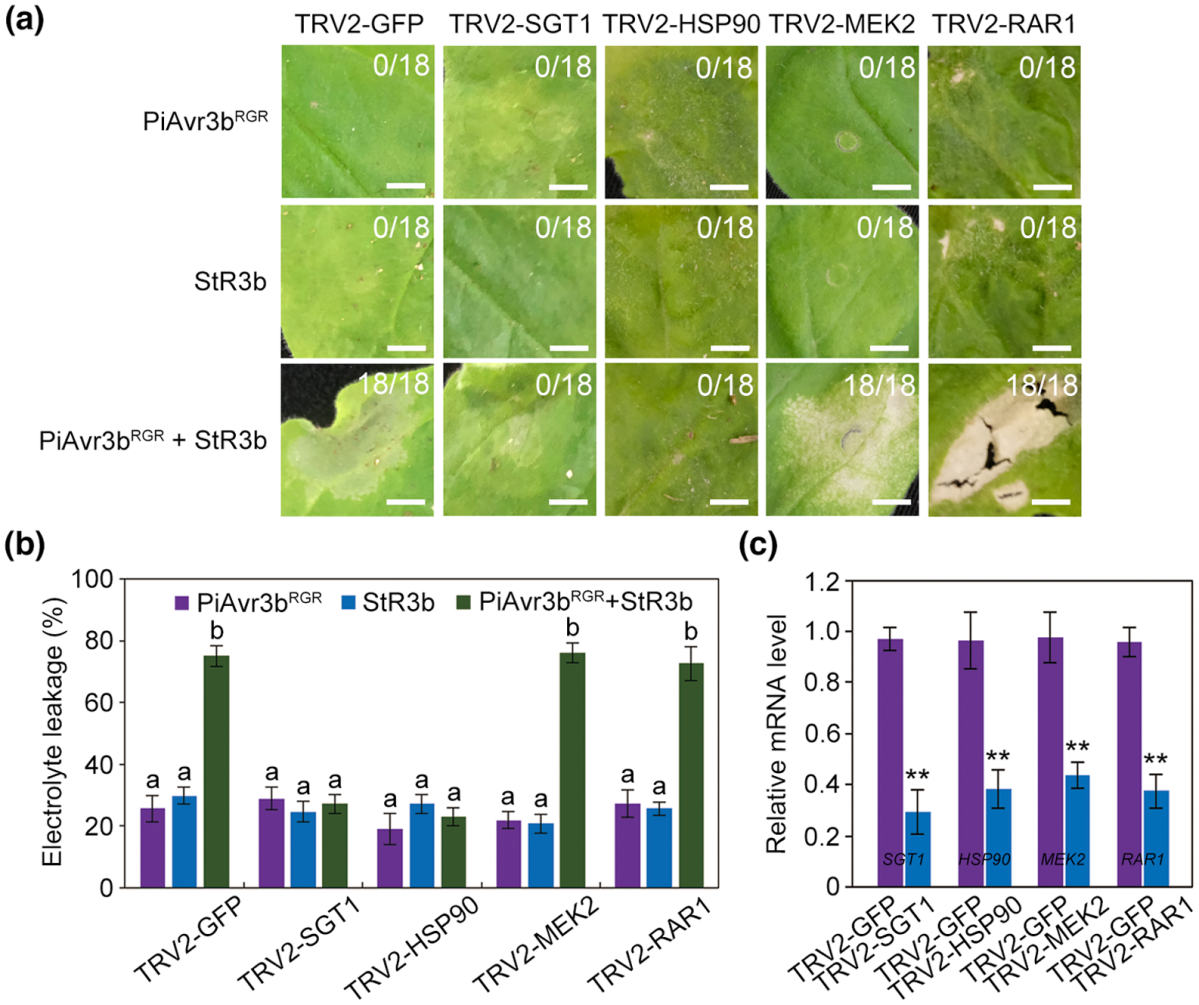
Effects of virus‐induced gene silencing (VIGS) of selected key plant immune components on the PiAvr3b and StR3b interactions in *Nicotiana benthamiana*. (a) Effects of silencing of *SGT1*, *HSP90*, *MEK2*, and *RAR1* on PiAvr3b‐StR3b triggered cell death. Green fluorescent protein (GFP) was used as a control. Symptoms were photographed 4 days postagroinfiltration. Ratios indicate the fraction of agroinfiltration sites forming necrosis. The experiment was performed three times with six plants for each TRV construct. Scale bars, 5 mm. (b) Quantifying *N. benthamiana* cell death in (a) by electrolyte leakage assay. Error bars indicate *SD*. Different letters indicate statistically different values (Kruskal‐Wallis and Dunn's tests; *p* < 0.05). (c) Relative expression of *NbSGT1*, *NbHSP90*, *NbMEK2*, and *NbRAR1* after VIGS. The Wilcoxon rank‐sum test was used for statistical analysis. Two asterisks indicate statistical difference (Student's *t* test, *p* < 0.01). These experiments were repeated three times with similar results.

To better understand the signalling pathway modulating PiAvr3b‐StR3b recognition, we employed a virus‐induced gene silencing (VIGS) strategy (Liu et al., 2002) to silence several critical genes involved in the plant immune response. *NbSGT1*, *NbHSP90*, *NbMEK2*, and *NbRAR1* were selected because these genes function as regulators of both PTI and ETI (Figure S6) (Yang et al., 2017). We found that silencing of *NbMEK2* and *NbRAR1* did not affect HR triggered by PiAvr3b^RGR^ and StR3b, while silencing of *NbHSP90* and *NbSGT1* abolished HR (Figure 3). These results indicate that PiAvr3b‐StR3b‐triggered cell death requires HSP90 and SGT1 but not *NbMEK2* and *NbRAR1*.

### 2.4 Both PiAvr3b isoforms promote *P. infestans* infection by suppressing PAMP‐ and effector‐triggered cell death

**FIGURE 4:**
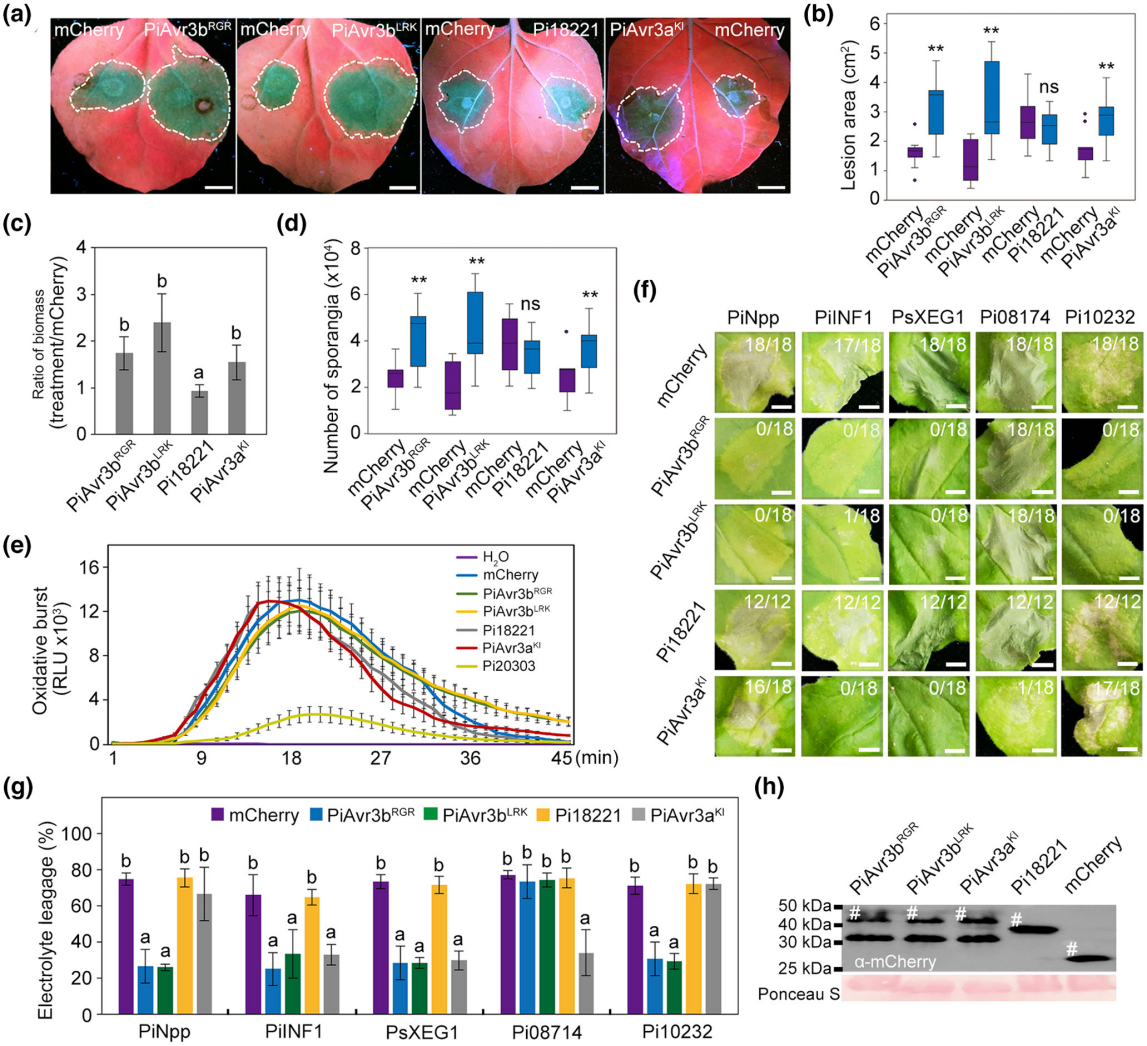
Expression of PiAvr3b isoforms in *Nicotiana benthamiana* enhances *Phytophthora infestans* infection and suppresses pathogen‐associated molecular pattern (PAMP)‐ and effector‐induced cell death. (a) Symptoms of *P. infestans* infecting *N. benthamiana* leaves that transiently expressed PiAvr3b isoforms. Pi18221 is a truncated paralog of PiAvr3b. All constructs shown were expressed as mCherry fusions. mCherry and PiAvr3a^KI^ served as controls. Pictures were taken 5 days after *P. infestans* zoospore inoculation. Dotted lines outline lesions. (b) Quantification of lesion sizes such as shown in (a). Pooled data from three independent experiments that comprise eight to 10 inoculations are presented as mean ± *SD*. Asterisks indicate significant differences (*p* < 0.01) based on the Wilcoxon rank‐sum test; ns, not significant. (c) Quantitative measurement of relative *P. infestans* biomass shown in (a). *P. infestans* biomass in *N. benthamiana* leaves expressing PiAvr3b isoforms, Pi18221, or PiAvr3a^KI^, was determined via quantitative PCR and normalized to the mCherry control. Kruskal‐Wallis and Dunn's tests were applied for statistical analysis, and letters indicate statistically different values (*p* < 0.01). (d) Numbers of sporangia isolated from diseased tissues shown in (a). The *y* axis shows sporangia numbers (per lesion) recovered at 6 days postinoculation from each treatment. Error bars depict *SD*. Two asterisks indicate sporangial numbers significantly different from the mCherry control; ns, not significant (Wilcoxon rank‐sum test, *p* < 0.01). (e) Reactive oxygen species production in *N. benthamiana* leaf discs overexpressing mCherry, PiAvr3b^RGR^, PiAvr3b^LRK^, Pi18221, PiAvr3a^KI^, or Pi20303 after treatments of 100 μM flg22 or water control. The relative luminescent units (RLU) were detected over a 45‐min period. Mean values (±*SD*) of eight replicates are shown. This experiment was repeated three times and showed similar results. (f) Cell death responses on *N. benthamiana* leaves co‐expressing PiAvr3b variants and *Phytophthora*‐originated PAMPs. *Agrobacterium* strains harbouring PiNpp, PiINF1, PsXEG1, Pi08174, or Pi10232 were co‐infiltrated with *Agrobacterium* strains expressing mCherry, PiAvr3b^RGR^, PiAvr3b^LRK^, Pi18221, or PiAvr3a^KI^ at a 1:1 ratio and a final OD_600_ of 0.4. Ratios on pictures indicate the agroinfiltrated areas that showed necrosis. Twelve or 18 agroinfiltration sites were photographed after 3–5 days. The experiment was repeated three times with similar results. Scale bars, 5 mm. (g) Quantification of cell death in (f) by electrolyte leakage assay. Electrolyte leakage from infiltration sites was measured as a percentage of leakage from boiled samples. Error bars represent *SD*. Two biological replicates were performed and showed similar results. Different letters indicate statistically different values (Kruskal–Wallis and Dunn's tests, *p* < 0.05). (h) Western blot with α‐mCherry antibody showing fusion protein expression in *N. benthamiana*. Protein loading is indicated by Ponceau S staining. Hash symbols indicate target proteins.

As previously mentioned, phylogenetic analyses identified two isoforms of PiAvr3b: PiAvr3b^RGR^ and PiAvr3b^LRK^. To further delineate their activities and roles in regulating plant defence, both PiAvr3b isoforms were fused to mCherry and transiently expressed in *N. benthamiana* by agroinfiltration. Compared to the mCherry control, both PiAvr3b^RGR^ and PiAvr3b^LRK^ produced approximately 2‐fold larger lesions, which was confirmed by quantitative biomass and sporangial measurements (Figure 4a–d). Pi18221 was not observed to be involved in virulence.

Next, we tested whether the two PiAvr3b isoforms could suppress the burst of reactive oxygen species (ROS) that plants deploy for defence against pathogens. We treated *N. benthamiana* leaf discs with bacterial flg22, which can induce an ROS burst, and found that neither PiAvr3b^RGR^ nor PiAvr3b^LRK^ suppressed ROS bursts, although the positive control Pi20303 (a paralogous effector of PiAvrblb2; Zheng et al., 2014) exhibited ROS suppression (Figure 4e). To further determine if either PiAvr3b isoform could inhibit PTI, we expressed three *Phytophthora* PAMPs (PiNpp, PiINF1, and PsXEG1) in *N. benthamiana* leaves. We found that both PiAvr3b^RGR^ and PiAvr3b^LRK^, but not Pi18221, suppressed necrosis induced by all three PAMPs; the positive control PiAvr3a^KI^ (Ma et al., 2015) suppressed only PiINF1‐ and PsXEG1‐associated necrosis (Figure 4d,f). We also tested the ability of PiAvr3b^RGR^ and PiAvr3b^LRK^ to suppress effector‐triggered cell death. We transiently expressed two necrosis‐inducing RxLR effectors (Pi08174 and Pi10232) (Wang et al., 2019) in *N. benthamiana* leaves, and identified that both PiAvr3b^RGR^ and PiAvr3b^LRK^ suppressed Pi10232‐ but not Pi08174‐induced cell death (Figure 4d,f). Western blot analysis confirmed expression of each effector protein in the leaves (Figure 4h). Thus, our findings suggest that both PiAvr3b isoforms enhance plant susceptibility by suppressing PAMP‐ and effector‐associated cell death.

### 2.5 Identification of residues in PiAvr3b that regulate plant defence

**FIGURE 5:**
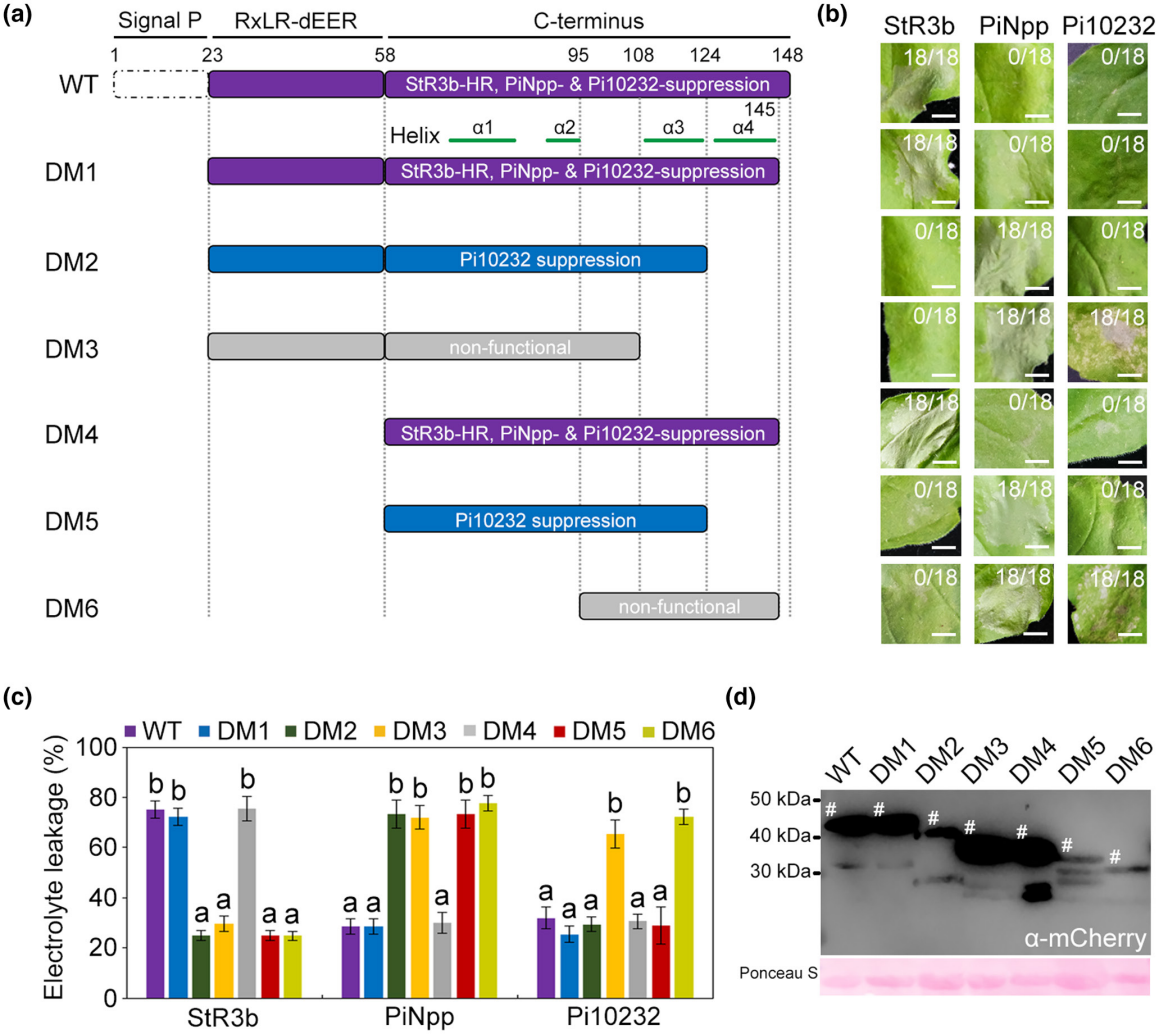
Mutational analysis of the PiAvr3b^RGR^ segments that contribute to StR3b‐mediated hypersensitive response (HR) and PiNpp‐ or Pi10232‐triggered cell death. (a) Schematics illustrating the six truncated PiAvr3b^RGR^ fragments (DM1–DM6) tested in the cell death assay. The dashed rectangle denotes the signal peptide (Signal P). The mature PiAvr3b^RGR^ (without signal P) was used as a wild‐type (WT) control. Green bars represent the predicted α‐helices at the C‐terminus of PiAvr3b using Jpred4 program (http://www.compbio.dundee.ac.uk/jpred/). Fragments that stimulated StR3b‐mediated HR and suppressed PiNpp‐ and Pi10232‐induced necrosis are coloured purple. PiAvr3b^RGR^ truncations that retained only the ability to suppress Pi10232‐induced cell death are coloured blue. Fragments coloured grey represent nonfunctional mutants. (b) Functional analysis of the mCherry‐tagged PiAvr3b^RGR^ truncations shown in (a) on *Nicotiana benthamiana* leaves expressing StR3b, PiNpp, or Pi10232. Pictures were taken 3–5 days postagroinfiltration. Ratios indicate the fraction of agroinfiltrated leaf areas that developed necrosis. Eighteen agroinfiltration sites were tested in each experiment; two independent experiments showed similar results. Scale bars, 5 mm. (c) Quantification of cell death in (b) by measuring electrolyte leakage. Electrolyte leakage from infiltration sites was measured as a percentage of leakage from boiled samples. Error bars represent the *SD*. Different letters at the tops of bars represent significant differences (Kruskal–Wallis and Dunn's tests, *p* < 0.05.). (d) Incubation with α‐mCherry shows fusion proteins expressed in *N. benthamiana*. Protein loading was indicated by Ponceau S staining.

To dissect the PiAvr3b residues responsible for regulating plant defence, we selected PiAvr3b^RGR^ as a test case because no functional variation was observed between the two PiAvr3b isoforms. We made six truncation mutants of PiAvr3b^RGR^ (DM1–DM6) (Figure 5a) and tested their ability to activate StR3b‐mediated HR and suppress cell death triggered by PiNpp or Pi10232. We found that the fragment containing residues 58–145 (DM4) was the smallest fragment that could initiate StR3b‐mediated HR and suppress PiNpp‐mediated necrosis. Fragments lacking residues 125–145 were only capable of suppressing Pi10232‐mediated cell death (Figure 5b–d).

**FIGURE 6:**
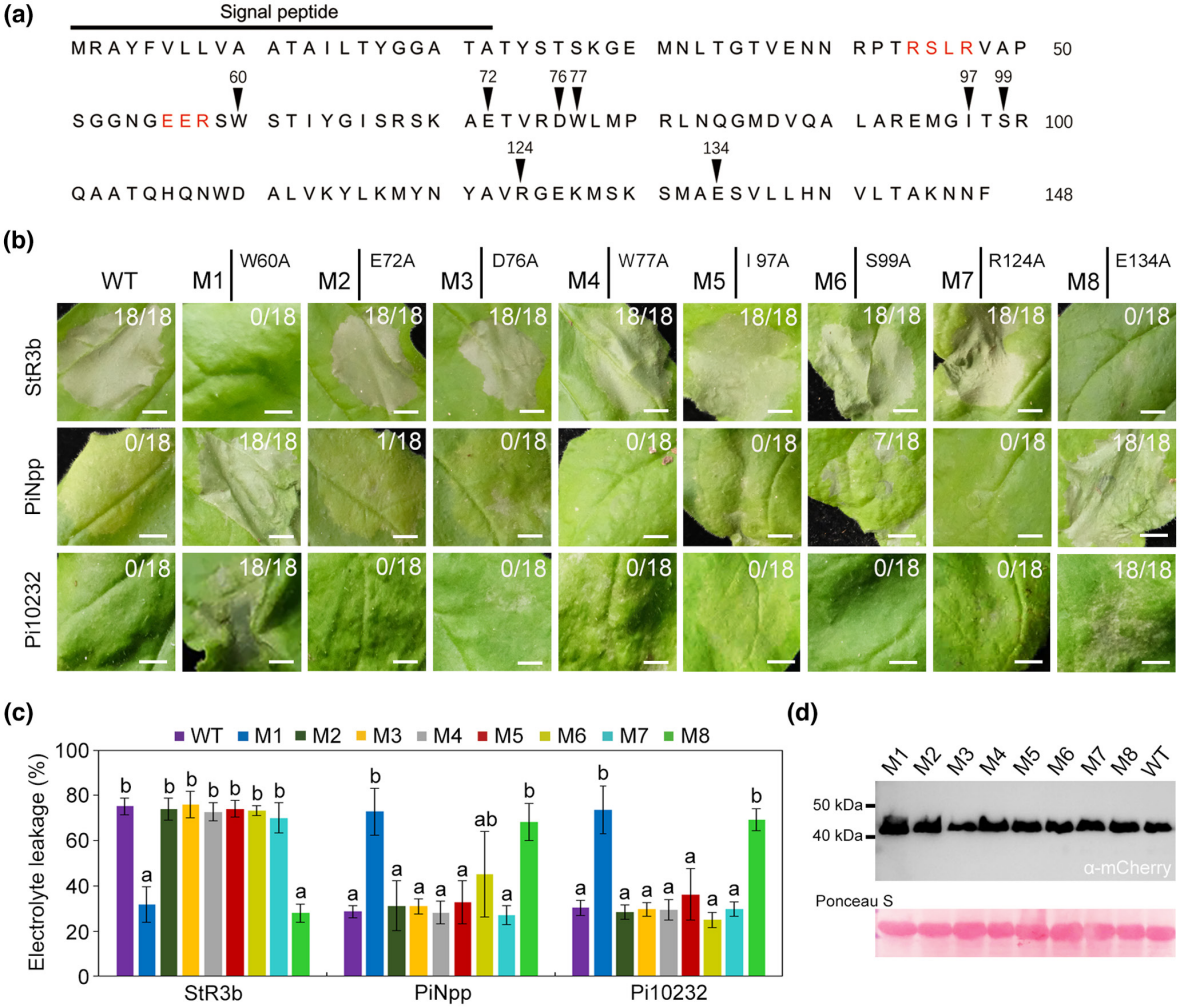
Mutational analysis of PiAvr3b^RGR^ residues involved in StR3b‐mediated activation and immune suppression. (a) The amino acid sequence of PiAvr3b^RGR^. The RxLR‐EER motif is highlighted in red. Black arrows and corresponding numbers indicate residues subject to alanine scanning. (b) *Nicotiana benthamiana* leaves showing cell death triggered by StR3b, PiNpp, or Pi10232 and alanine substitution mutants of PiAvr3b (M1‐M8). mCherry‐PiAvr3b^RGR^ (WT) was used as a control. Pictures were taken 3–5 days postagroinfiltration. Ratios indicate the fraction of agroinfiltrated leaf areas that developed necrosis. Eighteen agroinfiltration sites were included in each experiment; two independent experiments were performed and displayed similar results. Scale bars, 5 mm. (c) Quantification of cell death in (b) by measuring electrolyte leakage. Electrolyte leakage from infiltration sites was measured as a percentage of leakage from boiled samples. Error bars represent *SD*. Kruskal–Wallis and Dunn's tests were applied for statistical analysis with a *p*‐value cutoff <0.05. Different letters at the top of each bar represent significant differences. Two independent biological replicates were performed with similar results. (d) Immunoblot analysis of expressed fusion proteins using α‐mCherry. Protein loading was indicated by Ponceau S staining.

To further characterize the residues contributing to the dual activities of PiAvr3b, we performed a mutational analysis of PiAvr3b based on the identified sequence polymorphism (Figures 1 and 6a). When the conserved tryptophan was mutated (W60A), PiAvr3b could not stimulate StR3b‐mediated HR or suppress PiNpp‐ or Pi10232‐induced cell death (Figure 6b–d). In comparison, alanine substitution of glutamic acid (E72A), aspartic acid (D76A), tryptophan (W77A), isoleucine (I97A), or arginine (R124A) did not affect the dual activity of PiAvr3b. Intriguingly, we found that the mutation S99A partially attenuated suppression of the PiNpp‐triggered cell death but did not alter StR3b activation or Pi10232 suppression (Figure 6b,c). Next, we focused on the E134 residue, which was replaced by aspartic acid (E134D) in StR3b‐inactivating orthologs. We demonstrated that the E134 residue is also important for suppressing PiNpp‐ and Pi10232‐induced necrosis and activating StR3b‐mediated HR. Immunoblot confirmed that the PiAvr3b^RGR^ fusion proteins and alanine‐substitution mutants were intact and stable in *N. benthamiana* leaves (Figure 6d). These findings show that the C‐terminal effector domain mediates the dual activities of PiAvr3b. A single mutation of either the 60th or 134th amino acid led to the loss of StR3b activation and immune response suppression, suggesting that these two bioactivities are correlated.

### 2.6 Nuclear localization of PiAvr3b is critical for triggering HR and suppressing PTI, but not required for inhibiting effector‐induced necrosis

**FIGURE 7:**
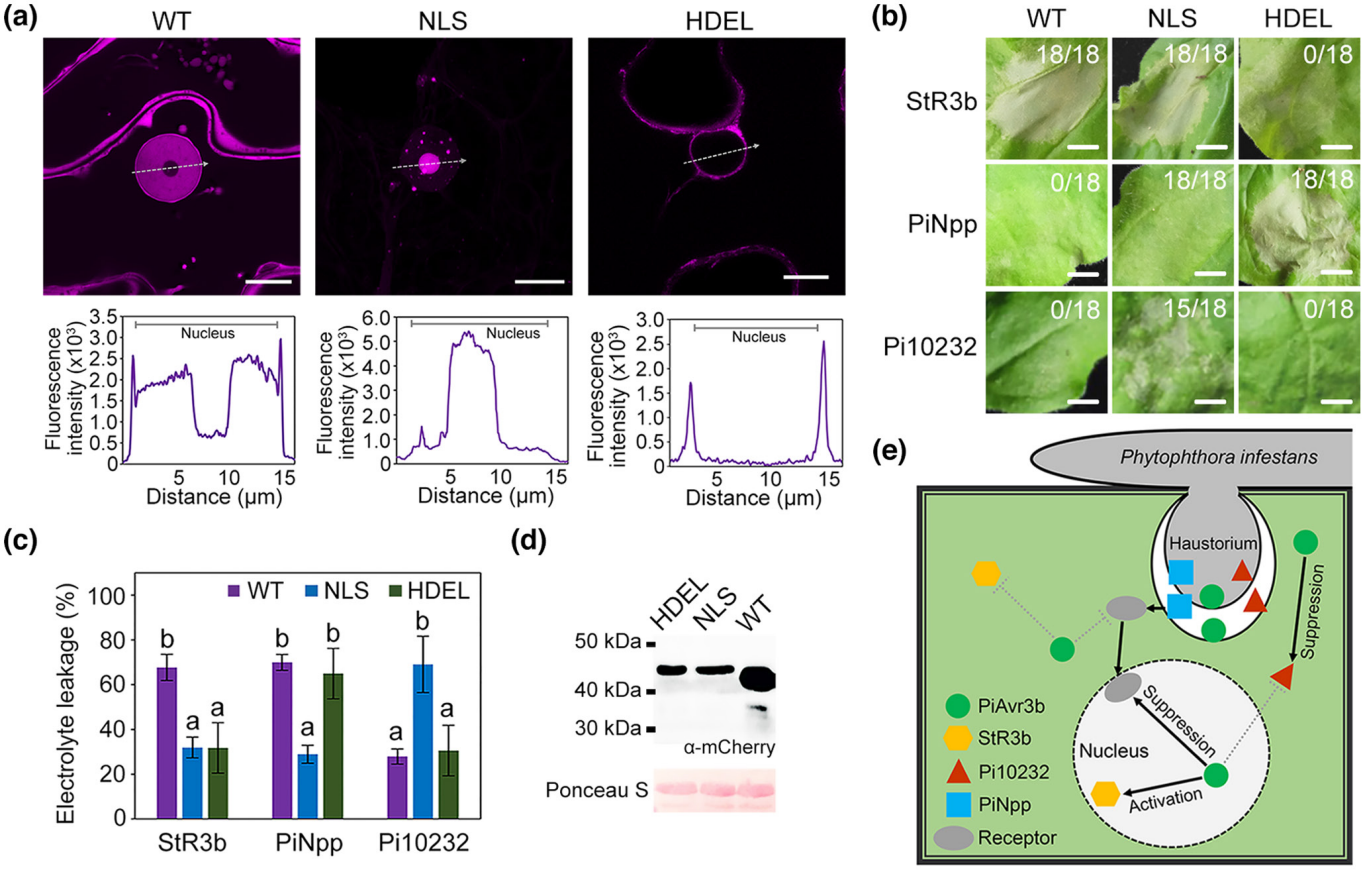
Mis‐targeting of PiAvr3b alters StR3b‐induced hypersensitive response (HR) and PiNpp‐ or Pi10232‐triggered cell death. (a) Subcellular localization of PiAvr3b^RGR^ fused with (mis)targeting signals in *Nicotiana benthamiana*. These include a nuclear localization signal (NLS) and an endoplasmic reticulum (ER) retention signal (HDEL), and mCherry‐PiAvr3b^RGR^ (WT) was used as a control. Images were captured at 2–3 days postagroinfiltration (dpa). Scale bars, 10 μm. The fluorescence density across the nucleus (grey bar) was measured and compared, as shown on the right panel. Three independent confocal microscopy observations (*n* > 18) were made, and representative images are shown. (b) *N. benthamiana* leaves showing cell death triggered by StR3b, PiNpp, or Pi10232 and mislocalization mutants of PiAvr3b. Photographs were captured at 3–5 dpa. Ratios between cell death phenotype and the total number of infiltrations (*n* = 18) are shown. The assays were repeated at least three times with similar results. (c) Quantification of cell death by measuring electrolyte leakage in (b). Electrolyte leakage from infiltration sites was measured as a percentage of leakage from boiled samples. Kruskal–Wallis and Dunn's tests were used for statistical analysis with a *p*‐value cutoff of 0.05. Letters indicate significant differences. Assays were repeated at least three times and showed similar results. (d) Immunodetection of mCherry‐PiAvr3b^RGR^ (WT), mCherry‐PiAvr3b^RGR^‐NLS (NLS), and mCherry‐PiAvr3b^RGR^‐HDEL (HDEL). Total protein from *N. benthamiana* leaves was analysed with α‐mCherry. Protein loading is indicated by Ponceau S staining. (e) Working model schematic showing the *Phytophthora infestans* RxLR effector PiAvr3b activates StR3b‐dependent HR and suppresses PiNpp‐ and effector‐triggered cell death.

Previous studies have shown that PiAvr3b localizes to both the nucleus and cytosol (Zheng et al., 2014). To examine whether subcellular localization determines the activity of PiAvr3b, we altered protein localization by fusing a nuclear localization signal (NLS) and an endoplasmic reticulum (ER) retention signal (signal peptide + HDEL) to the mCherry‐fused PiAvr3b^RGR^ generated previously, which formed PiAvr3b^RGR^‐NLS and PiAvr3b^RGR^‐HDEL. Employing high‐resolution confocal microscopy, we found that PiAvr3b^RGR^‐NLS predominantly localized in the nucleus, while PiAvr3b^RGR^‐HDEL accumulated in the ER and cytosol (Figures S7 and 7a). Relative to PiAvr3b^RGR^ and PiAvr3b^RGR^‐NLS, little fluorescent signal of PiAvr3b^RGR^‐HDEL was observed in the nucleus (Figure 7a). This observation was confirmed by colocalizing PiAvr3b^RGR^‐HDEL with ER markers, but not nuclear markers (Figure S8). In parallel, we tested the activity of these mislocalized proteins in cell death suppression and StR3b activation. As shown in Figure 7b,c, similar to PiAvr3b^RGR^, PiAvr3b^RGR^‐NLS triggered StR3b‐mediated HR and suppressed PiNpp‐induced cell death, whereas PiAvr3b^RGR^‐HDEL lost both activities. This implies that nuclear accumulation is required for PTI suppression and ETI induction of PiAvr3b^RGR^. Surprisingly, we found that Avr3b^RGR^‐NLS could not inhibit cell death induced by the RxLR effector Pi10232, while the ER‐localized PiAvr3b^RGR^‐HDEL remained capable of suppressing Pi10232‐induced necrosis (Figure 7b,c). Western blot analysis confirmed expression of these constructs in *N. benthamiana* (Figure 7d). These results imply that nuclear localization of PiAvr3b is indispensable for HR induction and PTI suppression activities, although it is not required for ETI suppression (Figure 7e).

## 3 DISCUSSION

In this study, we demonstrated genetic and transcriptional variation among *PiAVR3b* alleles in 95 naturally isolated *P. infestans* strains. PiAVR3b amino acid polymorphisms grouped the effector into two isoforms. We found that PiAvr3b protein sequences were only conserved in *Phytophthora* Clade I, and not all orthologs were capable of triggering cell death in the presence of the R protein StR3b. We found that HSP90 and SGT1 were required for PiAvr3b‐inducing cell death, and both PiAvr3b isoforms promoted *P. infestans* infection by suppressing different cell death inducers. Based on the mutational analysis, we identified distinct regions of PiAvr3b that determine its activity in cell death activation and plant immunity suppression. We also further illustrated that nuclear localization of PiAvr3b was critical for its HR elicitation and PTI inhibition activities, but not for suppression of effector‐induced necrosis.

Our results showed that gene deletion and silencing of PiAvr3b are responsible for evasion of StR3b‐mediated HR, which possibly contributed to the rise of super‐virulent *P. infestans* strains that overcame the *R1–R11* resistance genes in potatoes (Cooke et al., 2012; Fry, 2008; Tian et al., 2015). We found that PiAvr3b was only conserved across the six *P. infestans* sister species in the *Phytophthora* taxonomy Clade I, and not all of the PiAvr3b orthologs in these species could induce StR3b‐mediated cell death. Interestingly, other RxLR effectors with low sequence similarity, such as *P. sojae* Psoj292825 and *P. infestans* Pi19831, could trigger HR. This finding supports the view that effectors recognized by NLRs are likely to be functionally conserved and thus may be exploited in developing broad‐spectrum resistance (Lin et al., 2022). Based on the guard hypothesis, multiple effectors that target the same host protein do not need to be conserved in sequence to be recognized by the R protein guarding that target (Marathe & Dinesh‐Kumar, 2003). The presence of multiple PiAvr3b variants in diverse *Phytophthora* pathogens suggests that solanaceous crops maintain an active *StR3b*‐specific immune system, thus providing durable resistance to late blight disease. Multiple *NLR* genes, including *StR3b* in the wild potato (*S. pinnatisectum*), confer resistance to numerous prevalent races of *P. infestans* (Gu et al., 2020), suggesting that *StR3b* and its functional equivalents remain a useful resistance gene to control *Phytophthora* infection.

The broad recognition spectrum of StR3b illustrates the high level of complexity in *R* genes associated with the plant's immune surveillance system. In *N. benthamiana*, we found that the key ETI signalling components HSP90 and SGT1 were required for PiAvr3b‐StR3b‐induced HR. A similar observation was made for the interaction between R3a and PiAvr3a^KI^, in which both SGT1 and HSP90 are involved in the ETI response (Bos et al., 2006). Interestingly, HSP90 and SGT1 have been documented as essential regulators for INF1‐ and NIP‐induced cell death (Kanneganti et al., 2006), suggesting crosstalk between the PRR and NLR signalling pathways. In addition, PiAvr3b suppressed cell death triggered by the RxLR effector Pi22798, and its necrosis‐inducing activity also relied on SGT1 (Wang et al., 2017). In *Arabidopsis* and *N. benthamiana*, SGT1, HSP90, and RAR1 form chaperone complexes to regulate ETI (Shirasu, 2009), but our results showed that RAR1 was not necessary for StR3b‐mediated cell death. Mitogen‐activated protein kinases (MAPKs), such as MAPKKK and MEK2, are associated with PTI and ETI (Pedley & Martin, 2005). Silencing of MEK2 compromised the RxLR effector PsAvh238‐induced cell death in *N. benthamiana* (Yang et al., 2017). However, this protein was dispensable for StR3b‐associated HR, indicating that HR induced by the StR3b–PiAvr3b interaction may have a different downstream signalling pathway.

Extensive studies on *Phytophthora* effectors suggest that most RxLR effectors could enhance *P. infestans* colonization (Wang et al., 2019), and their activities are often associated with PTI suppression (Yin et al., 2017). PiAvr3b was previously reported to suppress flg22‐induced PTI and PiNpp‐triggered cell death in tomatoes (Zheng et al., 2014; Zuluaga et al., 2016). Conversely, we found that both PiAvr3b^RGR^ and PiAvr3a^KI^ failed to interrupt the flg22‐induced ROS burst in *N. benthamiana*. The discrepancy between these results may be due to differences in PTI components among diverse plants. Our results showed that PiAvr3b strongly suppressed the immune responses triggered by different PAMPs, such as PiINF1, PsXEG1, and PiNpp. As distinct plant immune receptors recognize these PAMPs, PiAvr3b probably inhibits a shared signalling pathway associated with plant immunity. The MAPK pathway is worthy of further examination because PiAvr3b suppressed flg22‐dependent posttranslational MAP kinase activation in tomatoes (Zheng et al., 2014). Moreover, we noticed that PiAvr3b suppressed cell death triggered by the effector Pi10232 but not Pi08174, while an opposite observation was made for PiAvr3a^KI^ (Figure 4f). This difference may be due to distinct signalling pathways targeted by PiAvr3a^KI^ and PiAvr3b (Whisson et al., 2016). To better understand the mode of action of PiAvr3b, further investigation may be needed to identify proteins that directly interact with PiAvr3b.

The integrity of the effector protein functional domain is critical for bioactivity. We found that Pi18221, the truncated paralog of Avr3b, could not elicit StR3b‐associated cell death (Figure 2a,b). This was consistent with our mutational assay, in which deletion of the PiAvr3b C‐terminal domain (residues 58–145) impaired StR3b activation. Our attempt to find a single residue that could separate the dual activities of PiAvr3b was unsuccessful. This suggests that the HR‐induction and necrosis‐suppression activities are linked, even though mutation of S99A in PiAvr3b partially compromised suppression of PiNpp‐associated necrosis. The same effector domain controlled the suppression of PiNpp‐induced cell death and activation of StR3b, indicating possible structural similarity between the potential interacting targets. Therefore, deletion or silencing of *PiAVR3b* could be the most effective way for *P. infestans* to escape host surveillance during pathogen–host coevolution.

A large portion of the RxLR effectors tested thus far were localized in plant nuclei or cytoplasm (Wang et al., 2019). However, the relationship between subcellular dynamics and the bioactivity of various RxLR effectors is less well illustrated. Studies have shown that nucleocytoplasmic transport of RxLR effectors PiAvr1 and PsAvh238 is required for PTI suppression or cell death induction (Du et al., 2015; Yang et al., 2017). Relocalization of PiAvr3a^KI^ from the cytosol to endosomes was associated with activation of the corresponding *R* gene during infection (Engelhardt et al., 2012). Transient expression assays demonstrated that PiAvr3b was distributed in both the *N. benthamiana* cytosol and nucleus (Zheng et al., 2014), and was not altered during infection. To clarify the correlation between the subcellular localization and bioactivities of PiAvr3b, we attempted to force PiAvr3b to accumulate solely in the cytoplasm by adding a nuclear export signal (NES) (Figures [Link], [Link]). However, we observed that PiAvr3b‐NES could still sufficiently trigger StR3b‐HR and suppress PiNpp‐necrosis (Figure S9). This result differs from a previous report that showed that NES‐tagged PiAvr3b was nonfunctional (Wang et al., 2022). One possible explanation for this difference could be that a single NES is insufficient to export all of the protein from the nucleus, and in fact we observed c.25% fluorescent signal that still resided in the nuclei (Figure S9a). Alternatively, we tried to exclude PiAvr3b from the nucleus by adding an ER retention signal (HDEL), and PiAvr3b^RGR^‐HDEL no longer activated ETI, suggesting that nuclear localization is required for StR3b recognition. Consistent with these findings, balanced nucleocytoplasmic transportation of the *P. infestans* effector Avr1 was reported to regulate the activation of *R1*‐mediated HR and suppression of CRN2‐induced cell death (Du et al., 2015).

Because of the sequence similarity between StR3a and StR3b, StR3b was predicted to accumulate in the host cytoplasm and has a resistance mechanism similar to R3a (Li et al., 2011). Our data provide new evidence supporting a mechanistic distinction between these two R proteins. Therefore, it is crucial to identify the host targets that mediate interactions between StR3b and PiAvr3b to understand how this resistance protein can recognize a variety of RxLR effectors. Because the same amino acid substitution compromises both StR3b‐activation and PiNpp‐suppression, it is logical to envision a model that involves interactions with a plant target protein guarded by StR3b. It also seems plausible that Pi19831 and other PiAvr3b variants bind to the plant target protein and thereby trigger R3b‐dependent HR. The well‐known PTI‐negative regulator RIN4 can be modified by many different bacterial Avr proteins and is, therefore, guarded by the resistance proteins RPM1 and RPS2 (Jones & Dangl, 2006). Our study may represent another example of a potential host protein associated with a filamentous plant pathogen effector and its corresponding NB‐LRR immune receptor.

## 4 EXPERIMENTAL PROCEDURES

### 4.1 Phylogenetic analysis

All PiAvr3b orthologs were identified by reciprocal BLAST searches. Alignment of the mature PiAvr3b protein sequences and its homologs was implemented by MEGA X software (v. 10.0.5) (Kumar et al., 2018). The phylogenetic neighbour‐joining (NJ) tree was constructed using the Poisson model with default settings (uniform rates, pairwise deletion, and 1000 bootstraps).

### 4.2 *P. infestans* culture and plant growth conditions

All *P. infestans* isolates used in this study (Table S1) were maintained on rye–sucrose agar at 18°C in the dark. Zoospores were prepared according to the method by Yin et al. (2017). The concentration of zoospores was adjusted to approximately 100/μl before inoculation. Ten microlitre zoospores suspension was inoculated on detached leaves of *N. benthamiana*. *N. benthamiana* was grown in a cultivation room under controlled growth conditions: 14 h light at 22°C/10 h dark at 20°C, 50% humidity, and approximately 200 μmol^−2^ s^−1^ light intensity.

### 4.3 Plasmid construction

The plasmids and primers used in the study are listed in Tables S4 and S5, respectively. The genes that encode the mature effectors (without signal peptides) PiAvr3b^RGR^ (PITG_18215), PiAvr3b^LRK^ (PITG_18215), Pi18221 (PITG_18221), Pi19831 (PITG_19831), Pi05910 (PITG_05910), Pi08174 (PITG_08174), Pi10232 (PITG_10232), Pi22798 (PITG_22798), Pi20303 (PITG_20303), Ppar05910 (PPTG_05910), Ppar13713 (PPTG_13713), and Psoj282925 (PHYSODRAFT_292825) were cloned by PCR using the genomic DNA (gDNA) from the corresponding *Phytophthora* species. The *PiAVR3b* orthologous genes (*PbetAVR3b‐1* [JAANHX010000435.1], *PbetAVR3b‐2* [JAANHX010000435.1], *PcatAVR3b* [WXXK01001367.1], *Pida370AVR3b* [RCMS01000029.1], *Pida376AVR3b* [RCMS01000029.1], and *Ppal37054* [PHPALM_37054]) were synthesized by Gencreate, Wuhan, China. All effector genes were cloned into the plasmid pMDC‐R (harbouring mCherry) or pMDC‐G (harbouring green fluorescent protein [GFP]) using the In‐Fusion HD Cloning Kit (Takara) according to manufacturer's instructions. The PiAvr3b^RGR^ truncations and mislocalization mutants were constructed using primers listed in Table S4. NLS and NES/nes were used as previously reported (Du et al., 2015; Yang et al., 2017). To generate the ER‐targeting mutants, the signal peptide of *N. benthamiana* endochitinase (SOL ID: Niben101Scf07491Ctg009) and the ER retention signal (HDEL) were PCR amplified and fused to mCherry‐PiAvr3b.

### 4.4 HR and cell death suppression tests

*Agrobacterium tumefaciens* GV3101 was used for the microinfiltration, and the plasmids harbouring desired genes were introduced into *A. tumefaciens* via the freeze–thaw method (Weigel & Glazebrook, 2006). Microinfiltration was carried out as follows. First, *A. tumefaciens* cells possessing the different plasmids were cultured overnight, harvested, and resuspended in infiltration buffer (10 mM MgCl_2_, 1 mM 2‐(*N*‐morpholino) ethanesulfonic acid pH 5.6, 150 μM acetosyringone) to an OD_600_ of 0.4. Next, *N. benthamiana* leaves were infiltrated with the *A. tumefaciens* cultures using a 1‐ml needleless syringe. Lesions were then scored visually 3–5 days after infiltration. Each assay was performed employing at least six plants, in which two or three leaves were infiltrated. Each experiment was repeated at least three times, and the data were analysed by Kruskal–Wallis and Dunn's multiple comparisons tests.

### 4.5 VIGS assays in *N. benthamiana*

pTRV (Liu et al., 2002) was used as the backbone plasmid for all the VIGS constructs, including pTRV1, pTRV2‐GFP, pTRV2‐HSP90, pTRV2‐SGT1, pTRV2‐MEK2, and pTRV2‐RAR1. *A. tumefaciens* GV3101 was used and adjusted to an OD_600_ of 0.8 for the agroinfiltration. pTRV1 and pTRV2 constructs were co‐infiltrated into the two primary leaves of a plant at the four‐leaf stage. The efficacy of gene silencing was examined via reverse transcription‐quantitative PCR (RT‐qPCR) using *Nbactin* (Table S4) as the reference gene.

### 4.6 Immunoblot analysis of transiently expressed proteins

Agroinfiltration of mCherry‐tagged RxLR effectors was conducted using 4‐ to 6‐week‐old *N. benthamiana* leaves. Forty‐eight to 72 h after agroinfiltration, 1‐cm^2^ leaf discs were excised and ground into fine powders in liquid nitrogen. Five hundred microlitres of lysis buffer (20 mM Tris–HCl [pH 7.5], 150 mM NaCl, 0.5% NP40, 1 mM phenylmethylsulfonyl fluoride, 1 mM EDTA) supplemented with 1% protease inhibitor cocktail was added to each pulverized plant sample. Lysates were centrifuged at 4°C, 18,759 × *g* for 10 min, and supernatants were transferred to fresh tubes. For each sample, 100 μl of supernatant was mixed with 100 μl of 2× loading buffer, heated at 95°C for 5 min, and kept on ice. Ten microlitres of the protein samples was separated via 10% SDS‐PAGE and transferred to a 0.45‐μm nitrocellulose membrane (Cytiva) at 10 V for 1–2 h. Skimmed milk 5% (wt/vol) was used to block the membrane. Mouse monoclonal mCherry antibody (Abcam) was used as the primary antibody at 1:10,000 dilution. The membrane was washed with phosphate‐buffered saline with 0.1% Tween 20 before adding the secondary goat anti‐mouse immunoglobulin (IgG) horseradish peroxidase conjugate (Abcam) at 1:2000 dilution. The SuperSignal West Femto substrate (Thermo Scientific) for protein detection was used according to the manufacturer's instructions.

### 4.7 RT‐qPCR

Total RNA was isolated from infected *N. benthamiana* leaves with the RNeasy Plant Mini Kit (Qiagen). Total RNA (1 μg) was used for reverse transcription. First‐strand cDNA was synthesized using SuperScript III (Thermo Fisher) and qPCR was conducted employing the SYBR Premix Ex Taq II kit (Takara) according to the manufacturer's instructions.

### 4.8 *P. infestans* inoculation, biomass assay, and sporangial measurement

To evaluate the gene's function, *N. benthamiana* leaves were detached and transferred to a Petri dish 24 h after agroinfiltration. Infiltrated regions were inoculated with 10 μl of *P. infestans* Pi009 zoospores suspension (10^5^ zoospores/ml) and incubated at 25°C in the dark as previously described (Gu et al., 2020). Plants were photographed under high‐intensity UV light (Analytic Jena) at 5 days postinoculation. Infected areas on the leaves were quantified by ImageJ. Three independent biological replicates were included. To perform biomass measurement, total gDNA was extracted using a hexadecyltrimethylammonium bromide method (May & Ristaino, 2004) and quantified by 1% agarose gel electrophoresis. Quantitative PCR of gDNA was performed using CFX Connect (BioRad) as described by Gu et al. (2021) to obtain biomass measurements. Genomic DNA abundance or relative gene expression was measured by a 2^−ΔΔCt^ method using the *Actin* genes from both *P. infestans* and *N. benthamiana* as endogenous controls. To count sporangial numbers, whole leaves displaying lesions were immersed in 3 ml of water and vortexed vigorously. The number of sporangia harvested from each leaf was counted using a light microscope (BX63; Olympus).

### 4.9 Measurement of ROS

ROS production was examined using a luminol/peroxidase‐based method (Albert et al., 2015). Briefly, 0.4 cm leaf discs were collected from 6‐week‐old *N. benthamiana* leaves and floated on 200 μl of sterile water (using 96‐well plates) overnight. The following day, water was replaced with the luminol (35.4 μg/ml)/peroxidase (10 μg/ml) reaction solution (dissolved in sterile water), and 100 nM of the flagellin‐derived peptide flg22 (Genscript Biotech Corp.) or an empty vector. Luminescence was measured by the Varioskan LUX microplate reader (Thermo Fisher Scientific).

### 4.10 Electrolyte leakage assay

An electrolyte leakage assay was conducted to evaluate PAMP‐ or effector‐triggered cell death, according to a previously described method (Yang et al., 2017). Briefly, for each sample, five leaf discs (1 cm in diameter) from agroinfiltrated areas were collected and floated on 5 ml of distilled water overnight at 25°C. The next day, the conductivity of the bathing solution was measured using a conductivity meter (FE32 FiveEasy; Mettler Toledo), which yielded value A. The leaf discs in the bathing solutions were boiled in sealed tubes for 30 min. After cooling the solution to 25°C, the conductivity was measured again to obtain value B. Ion leakage was measured as a percentage of total ions: (value A/value B) × 100. In the case of agroinfiltration, the ratio of total ions was compared using the Wilcoxon rank‐sum test. Multiple independent measurements were compared by Kruskal–Wallis and Dunn's multiple comparisons tests.

### 4.11 Confocal microscopy

The laser scanning confocal microscope LSM 900 with Airyscan 2 (Zeiss) was used to examine the expression and subcellular localization of GFP‐ or mCherry‐fused proteins in *N. benthamiana* leaves. Images were captured using a 40× water objective lens with excitation/emission settings of 561 nm/570–630 nm for mCherry and 488 nm/495–555 nm for GFP under super‐resolution. Single optical section images were acquired from leaf epidermal cells, and *z*‐stacks were collected from leaf cells and projected and processed using the Carl Zeiss ZEN blue 3.2. Images were edited by Adobe Photoshop CS5 v. 12.0.1.

### 4.12 Yeast two‐hybrid assay

The yeast two‐hybrid assay was conducted using the Matchmaker system (Clontech). Briefly, the bait plasmid pGBKT7‐PiAvr3b^RGR^ and the prey plasmid pGADT7‐StR3b were introduced into *Saccharomyces cerevisiae* AH109, and nutritional selection (triple dropout [TDO]/−Trp−Leu−His and quadruple dropout [QDO]/−Trp−Leu−His−Ade) was used to recover transformants. The pGBKT7‐53 and pGADT7‐T plasmid pair was used as a positive control, and the empty plasmids pGBKT7 and pGADT7 were applied to rule out self‐activation of the reporter genes.

## CONFLICT OF INTEREST

The authors declare that they have no conflict of interest.

## Supporting information


**Figure S1** Genotypic and transcriptional analysis of *PiAVR3b* alleles in 95 isolates. Lanes 1–95 represent isolates 1–95. Lane 96 is the water control. (a) PCR amplification of the *PiAVR3b* ORF. (b) PCR amplification of endogenous control *PiActin*. (c) Reverse transcription (RT)‐PCR amplification of the *PiAVR3b* ORF. (d) RT‐PCR amplification of endogenous control *PiActin*.
[Correction added on 20 February 2023, after first online publication: Table S1 in the Supporting Information has been corrected in this version]

**Figure S2** Sequence alignment of PiAvr3b and Pi18221. (a) Alignment of *PiAVR3b* and *Pi18221* nucleotide sequences. The purple rectangle denotes the two nucleotides missing in *Pi18221*. (b) Alignment of PiAvr3b and Pi18221 amino acid sequences. The blue rectangle indicates the RxLR motifs.

**Figure S3** Transcriptional analysis of *Pi18221* in the *Phtyptophthora infestans* isolates that harbor *PiAVR3b*. Lanes are numbered in the same order as in Figure S1c.

**Figure S4** Western blot detection of mCherry‐fused PiAvr3b homologs. Total protein was extracted 2‐3 days after agroinfiltration (dpa) in *Nicotiana benthamiana leaves*. Protein loading is indicated by Ponceau stain (Ponceau S).

**Figure S5** Yeast two‐hybrid assay to identify PiAvr3b‐StR3b interactions. All yeast transformants were grown on DDO (‐Leu, ‐Trp) medium; only the positive control (BD‐p53 and AD‐T) grew on TDO (‐Leu, ‐Trp, ‐His) and QDO (‐Leu, ‐Trp, ‐His, ‐Ade) media. BD‐EV and AD‐EV are the empty bait and prey vectors, respectively.

**Figure S6** Schematics showing the fragments within *GFP*, *NbSGT1*, *NbHSP90*, *NbMEK2*, and *NbRAR1* (purple rectangles) used to generate the VIGS constructs and reverse transcription‐quantitative PCR primers (blue rectangles) employed to detect gene silencing. Scale bar, 100 bp.

**Figure S7** Confocal microscopy showing *Nicotiana benthamiana* leaves with transient expression of NLS‐ and HDEL‐tagged mCherry‐PiAvr3bRGR. White arrows indicate nuclei. Scale bar, 20 μm. Two independent confocal microscopy observations (n > 12) were made, and representative projection images were shown.

**Figure S8** Co‐localization of NLS‐ or HDEL‐tagged mCherry‐PiAvr3bRGR with a nuclear marker (GFP‐NLS) (a) or ER marker (GFP‐HDEL) (b) in *Nicotiana benthamiana* leaves. White arrows indicate nuclei. Scale bar, 10 μm. Three independent confocal microscopy observations (n > 18) were made, and representative projection images were shown.

**Figure S9** NES‐tagged PiAvr3bRGR fails to alter StR3b‐induced hypersensitive response and PiNp‐ or Pi10232‐triggered cell death. (a) Subcellular localization of PiAvr3bRGR fused with an N‐terminal nuclear export signal (NES) or a null functional form (nes) in *Nicotiana benthamiana*. Images were captured at 2‐3 days postagroinfiltration (dpa). Scale bar, 10 μm. Fluorescence density across the cytoplasm and nucleus (grey bar) was measured and compared, as shown on the right panel. Three independent confocal microscopy observations (n > 18) were made, and representative images were shown. (b) Western blot analysis of expressed fusion proteins with α‐mCherry. Protein loading was indicated by Ponceau S staining. (c) *N. benthamiana* leaves showing cell death triggered by StR3b, PiNpp, or Pi10232 and mislocalization mutants of PiAvr3b. Photographs were captured at 3–5 dpa. The relationship between the cell death phenotype and the total number of infiltrations (n = 18) is shown. Assays were repeated at least three times with similar results. (d) Quantification of cell death by measuring electrolyte leakage in (c). Electrolyte leakage from infiltration sites was measured as a percentage of leakage from boiled samples. Kruskal‐Wallis test was used for statistical analysis with a *p*‐value cutoff of 0.05. Letters at the top of each bar indicate statistically significant differences.

**Figure S10** Confocal microscopy showing *Nicotiana benthamiana* leaves with transient expression of NES‐ and nes‐tagged mCherry‐PiAvr3bRGR. White arrows indicate nuclei. Scale bar, 20 μm. Two independent confocal microscopy observations (n > 12) were made, and representative projection images were shown.

**Figure S11** Co‐localization of NES‐tagged mCherry‐PiAvr3bRGR and GFP‐Pi22798 in *Nicotiana benthamiana* leaves. n: nucleus; arrow indicates nucleolus. Scale bar, 10 μm. Two independent confocal microscopy observations (n > 18) were made, and representative images were shown. The fluorescence density across the cytoplasm and nucleus was measured and compared, as shown on the bottom right panel.

**Table S1** *Phytophthora infestans* isolates used in this study and polymorphism data associated with PiAvr3b.

**Table S2** Genetic diversity in PiAvr3b of *Phytophthora infestans* populations sampled from China before 2016 and after 2018.

**Table S3** List of *PiAVR3b* orthologous genes or *PiAVR3b‐ike* genes in different *Phytophthora* species. The absence and presence of *PiAVR3b* polymorphisms in both genomic DNA and cDNA are also listed.

**Table S4** Primers used in this study.

**Table S5** Constructs used in this study.

## Data Availability

The data that support the findings of this study are available on request from the corresponding author.
