# Supplementary figures and images for: A single region of the Phytophthora infestans avirulence effector Avr3b functions in both cell death induction and plant immunity suppression
Source: Mol Plant Pathol. 2023 Jan 25;24(4):317–30. doi: 10.1111/mpp.13298 (PMC10013827; doi:10.1111/mpp.13298)

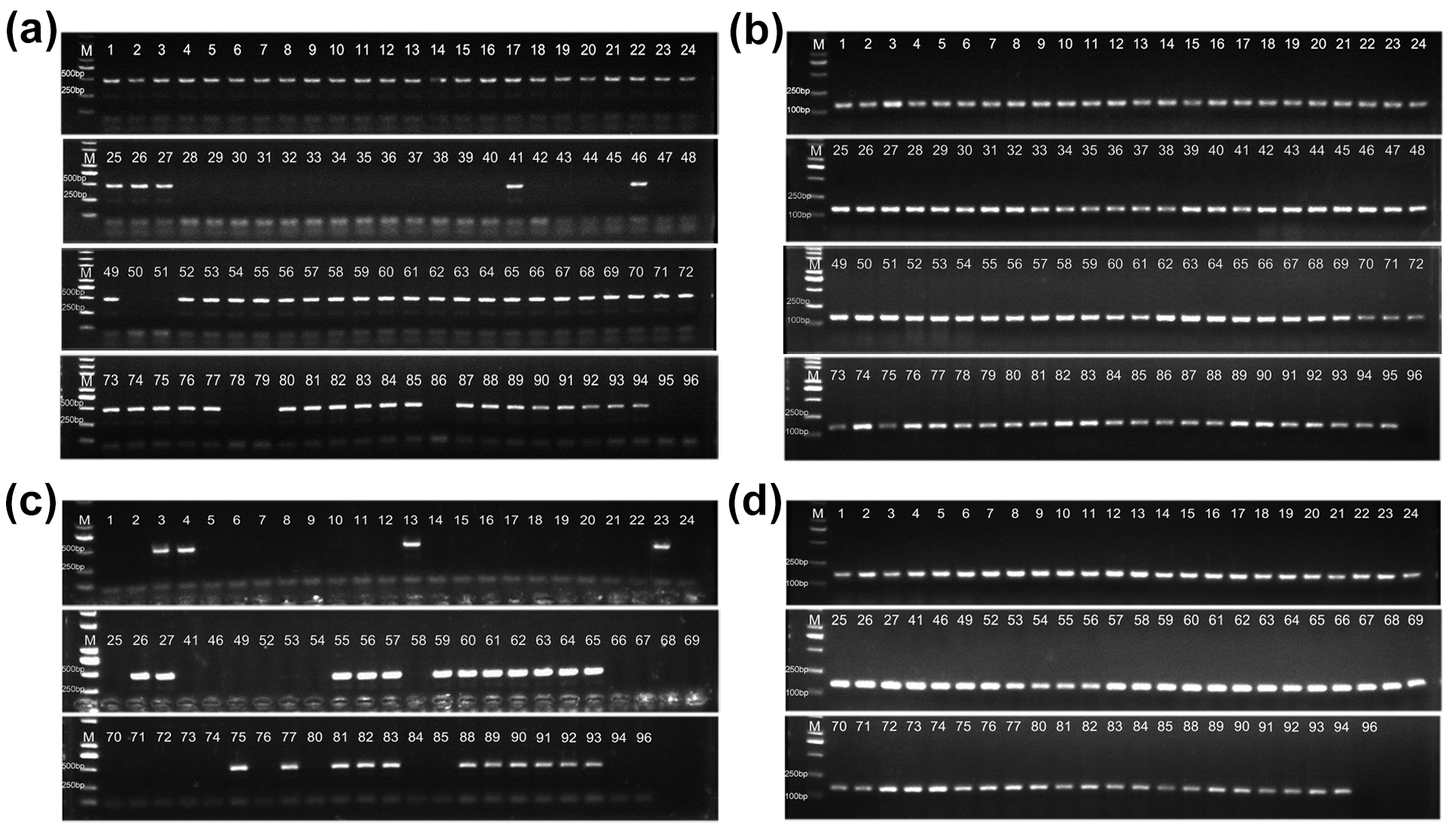

Supplement: Supplementary file 1 — Figure S1 Genotypic and transcriptional analysis of PiAVR3b alleles in 95 isolates. Lanes 1–95 represent isolates 1–95. Lane 96 is the water control. (a) PCR amplification of the PiAVR3b ORF. (b) PCR amplification of endogenous control PiActin. (c) Reverse transcription (RT)‐PCR amplification of the PiAVR3b ORF. (d) RT‐PCR amplification of endogenous control PiActin. [Correction added on 20 February 2023, after first online publication: Table S1 in the Supporting Information has been corrected in this version] [file MPP-24-317-s001.tif]

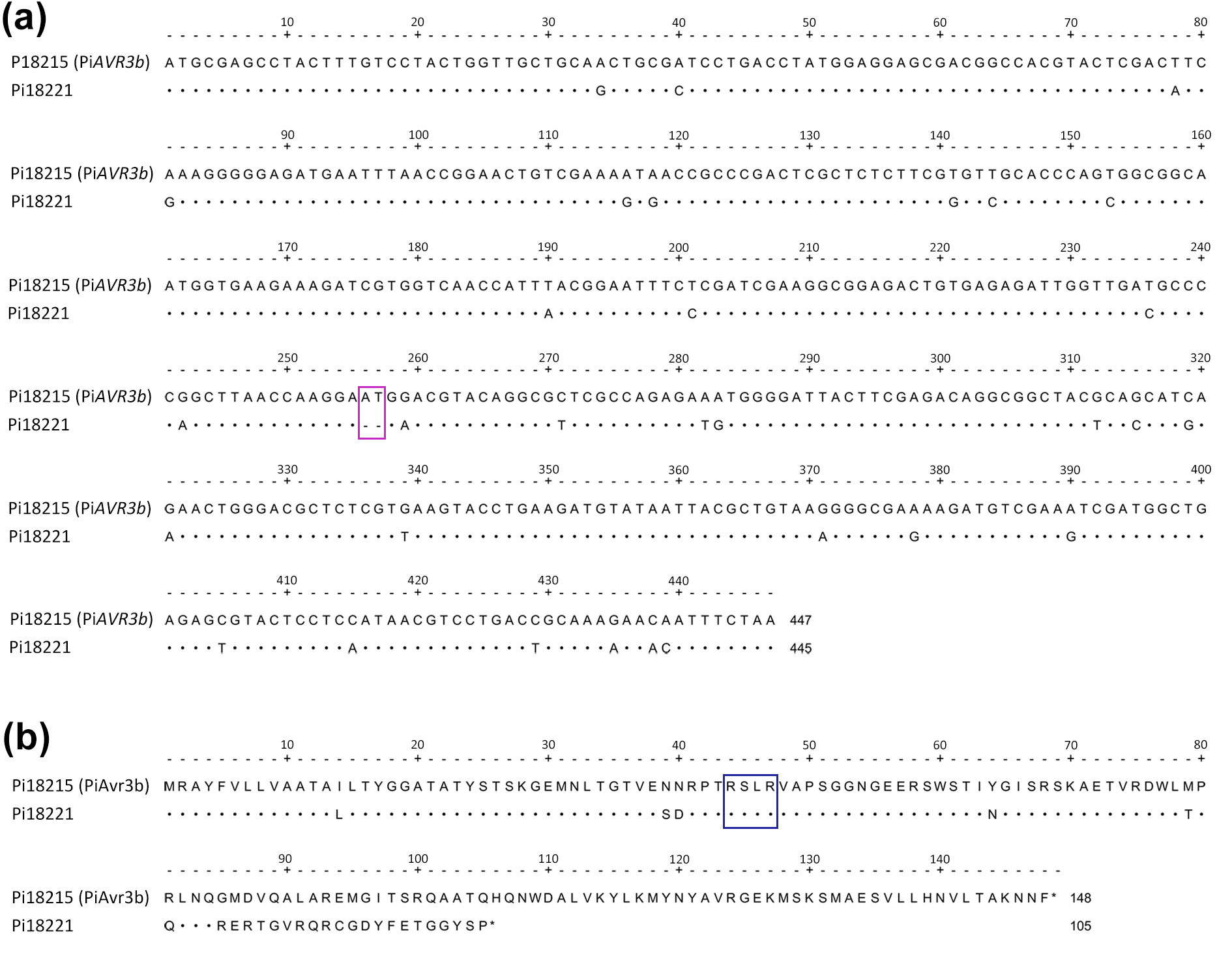

Supplement: Supplementary file 2 — Figure S2 Sequence alignment of PiAvr3b and Pi18221. (a) Alignment of PiAVR3b and Pi18221 nucleotide sequences. The purple rectangle denotes the two nucleotides missing in Pi18221. (b) Alignment of PiAvr3b and Pi18221 amino acid sequences. The blue rectangle indicates the RxLR motifs. [file MPP-24-317-s013.tif]

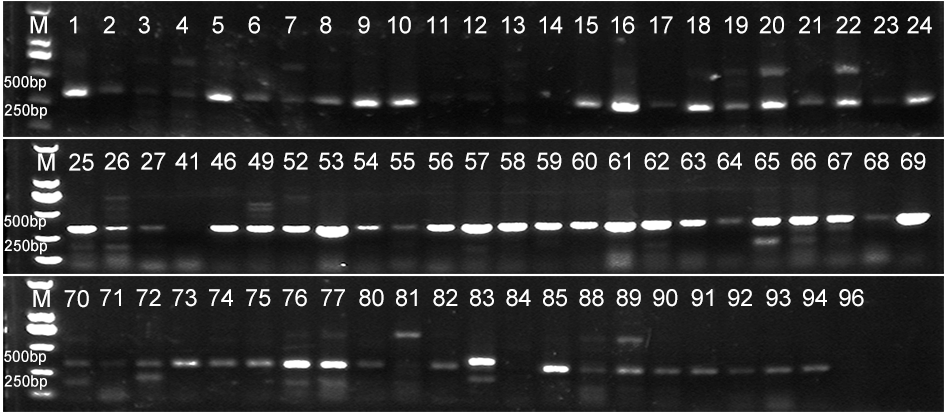

Supplement: Supplementary file 3 — Figure S3 Transcriptional analysis of Pi18221 in the Phtyptophthora infestans isolates that harbor PiAVR3b. Lanes are numbered in the same order as in Figure S1c. [file MPP-24-317-s016.tif]

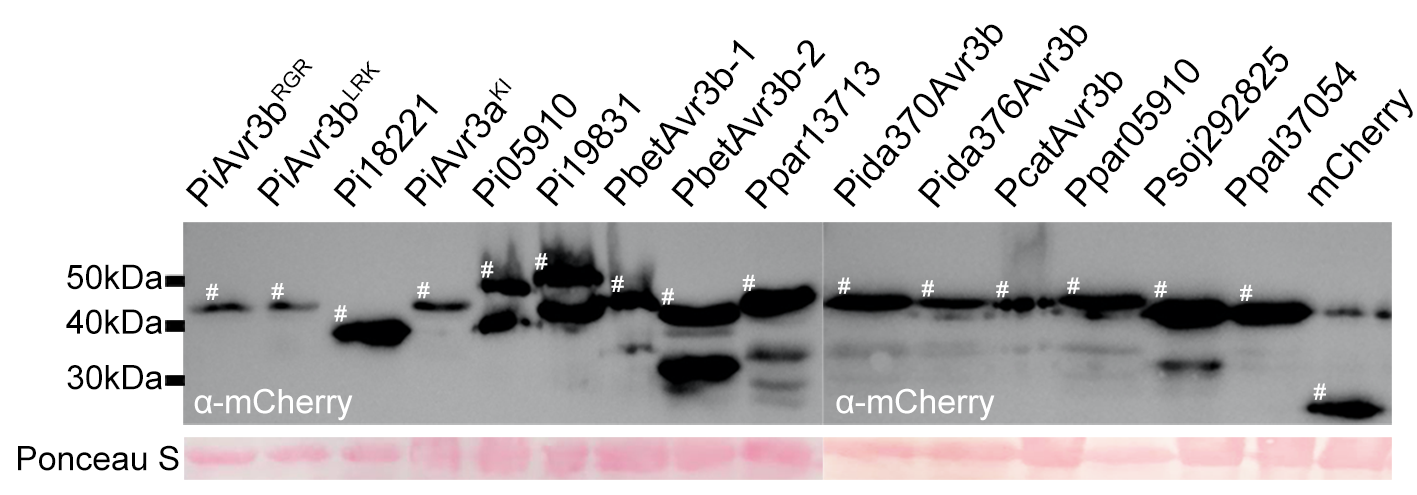

Supplement: Supplementary file 4 — Figure S4 Western blot detection of mCherry‐fused PiAvr3b homologs. Total protein was extracted 2‐3 days after agroinfiltration (dpa) in Nicotiana benthamiana leaves. Protein loading is indicated by Ponceau stain (Ponceau S). [file MPP-24-317-s010.tif]

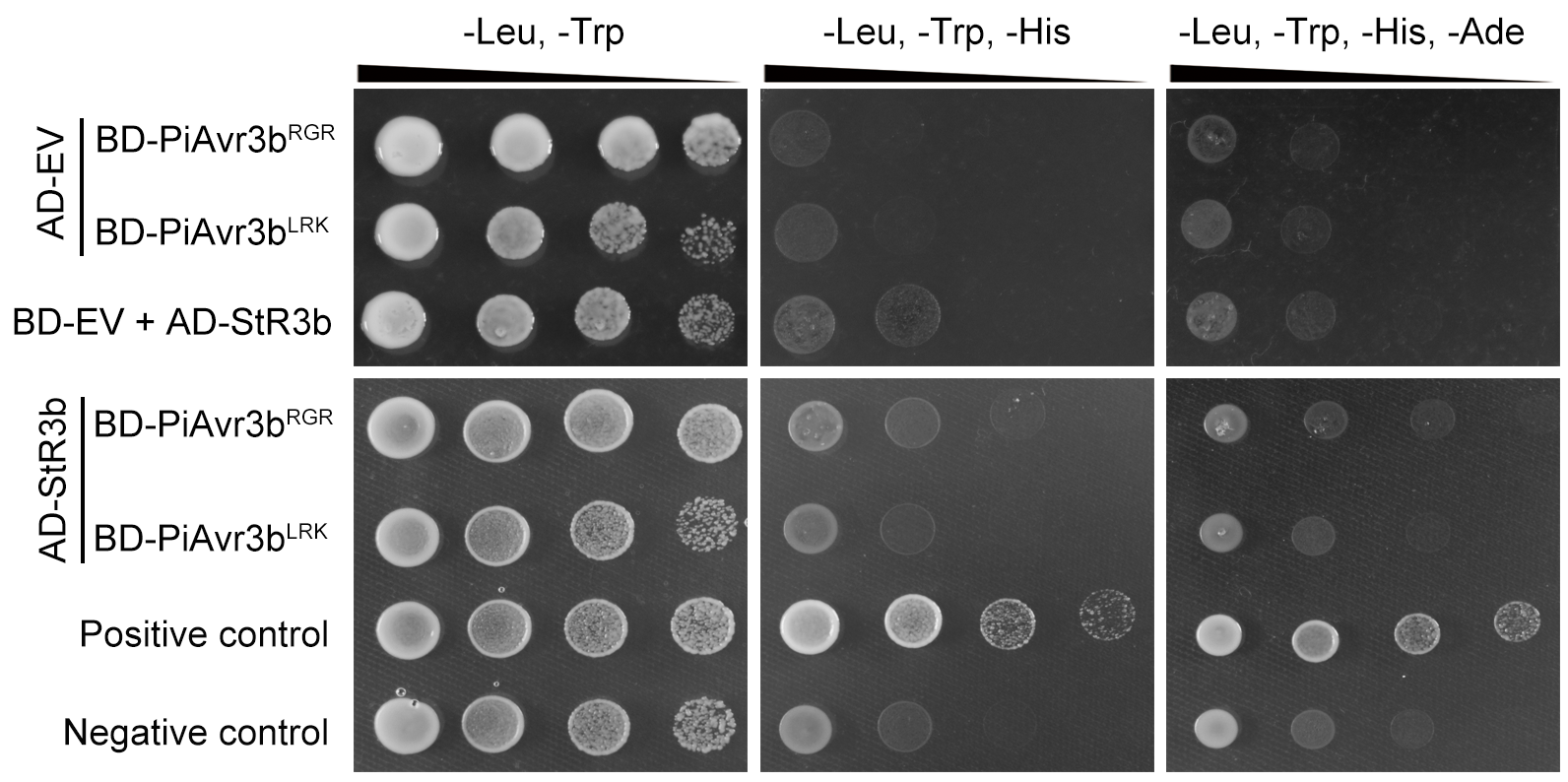

Supplement: Supplementary file 5 — Figure S5 Yeast two‐hybrid assay to identify PiAvr3b‐StR3b interactions. All yeast transformants were grown on DDO (‐Leu, ‐Trp) medium; only the positive control (BD‐p53 and AD‐T) grew on TDO (‐Leu, ‐Trp, ‐His) and QDO (‐Leu, ‐Trp, ‐His, ‐Ade) media. BD‐EV and AD‐EV are the empty bait and prey vectors, respectively. [file MPP-24-317-s006.tif]

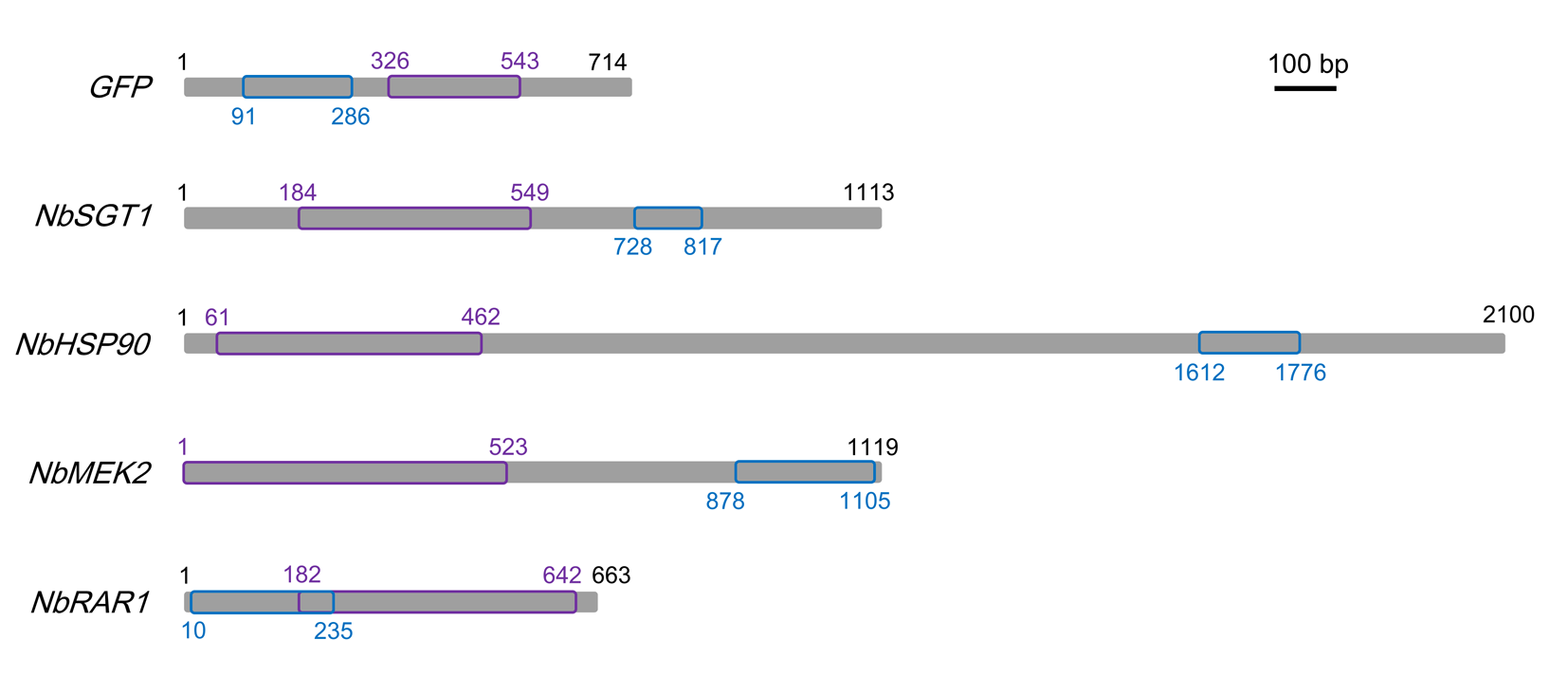

Supplement: Supplementary file 6 — Figure S6 Schematics showing the fragments within GFP, NbSGT1, NbHSP90, NbMEK2, and NbRAR1 (purple rectangles) used to generate the VIGS constructs and reverse transcription‐quantitative PCR primers (blue rectangles) employed to detect gene silencing. Scale bar, 100 bp. [file MPP-24-317-s003.tif]

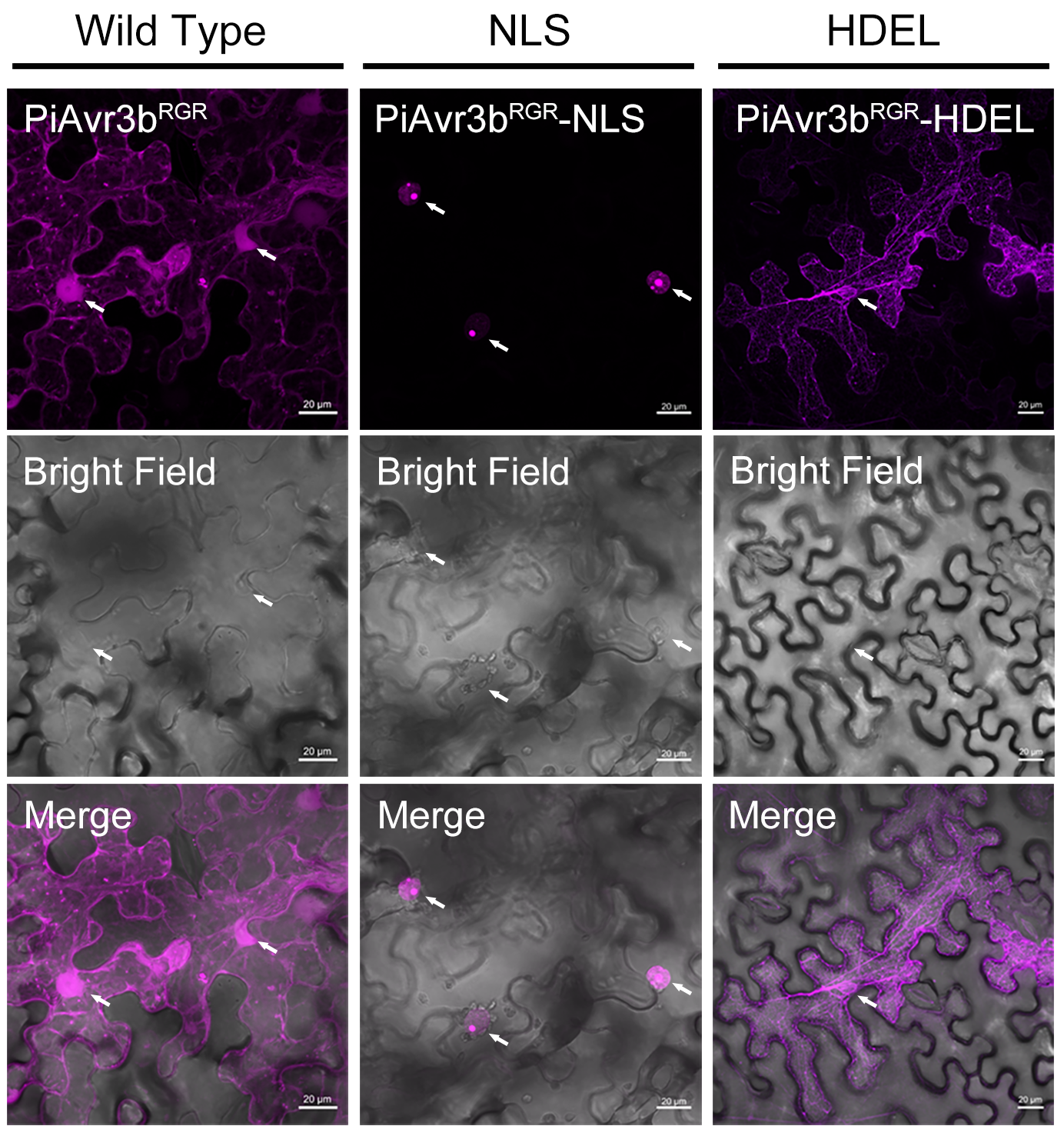

Supplement: Supplementary file 7 — Figure S7 Confocal microscopy showing Nicotiana benthamiana leaves with transient expression of NLS‐ and HDEL‐tagged mCherry‐PiAvr3bRGR. White arrows indicate nuclei. Scale bar, 20 μm. Two independent confocal microscopy observations (n > 12) were made, and representative projection images were shown. [file MPP-24-317-s009.tif]

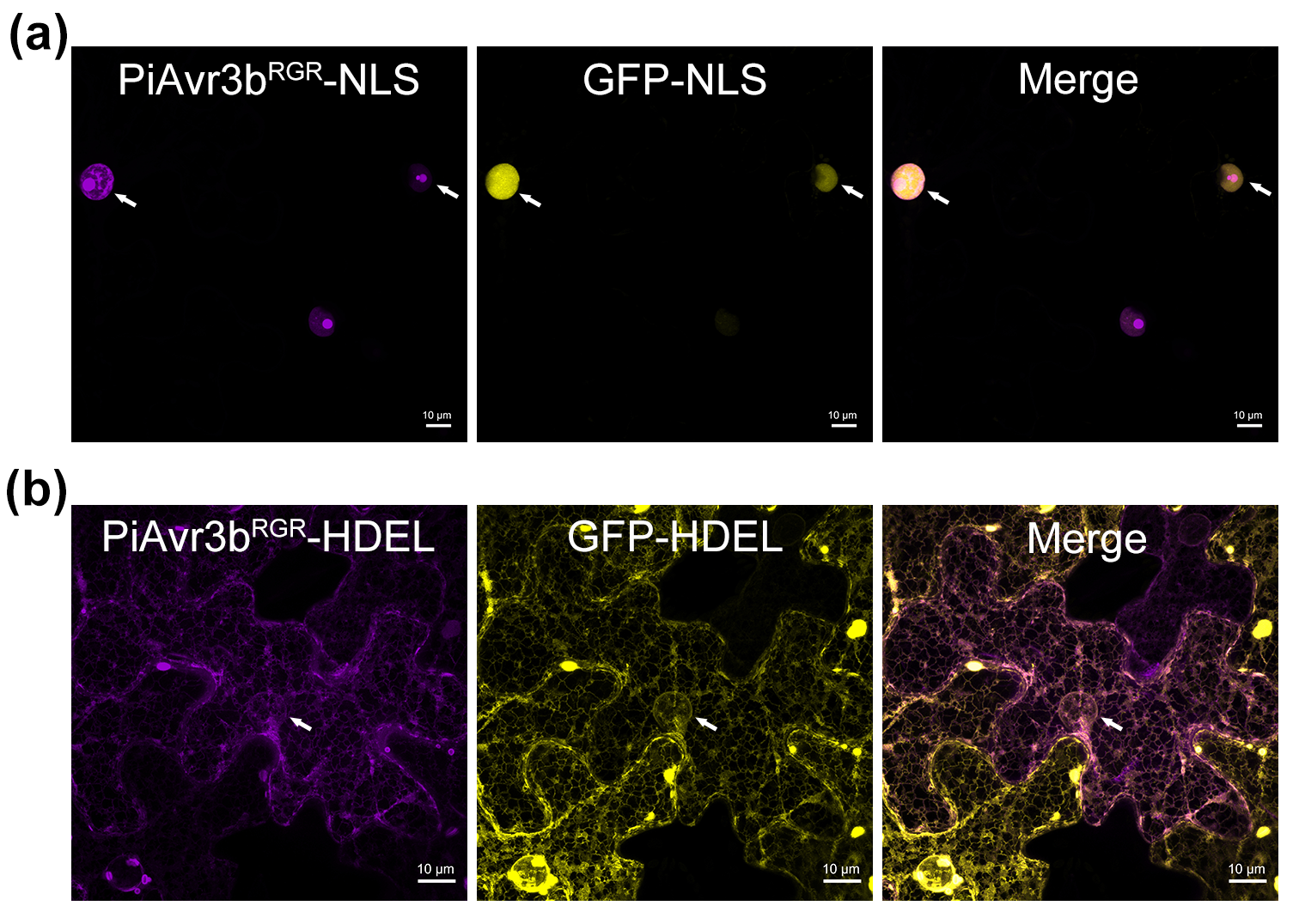

Supplement: Supplementary file 8 — Figure S8 Co‐localization of NLS‐ or HDEL‐tagged mCherry‐PiAvr3bRGR with a nuclear marker (GFP‐NLS) (a) or ER marker (GFP‐HDEL) (b) in Nicotiana benthamiana leaves. White arrows indicate nuclei. Scale bar, 10 μm. Three independent confocal microscopy observations (n > 18) were made, and representative projection images were shown. [file MPP-24-317-s002.tif]

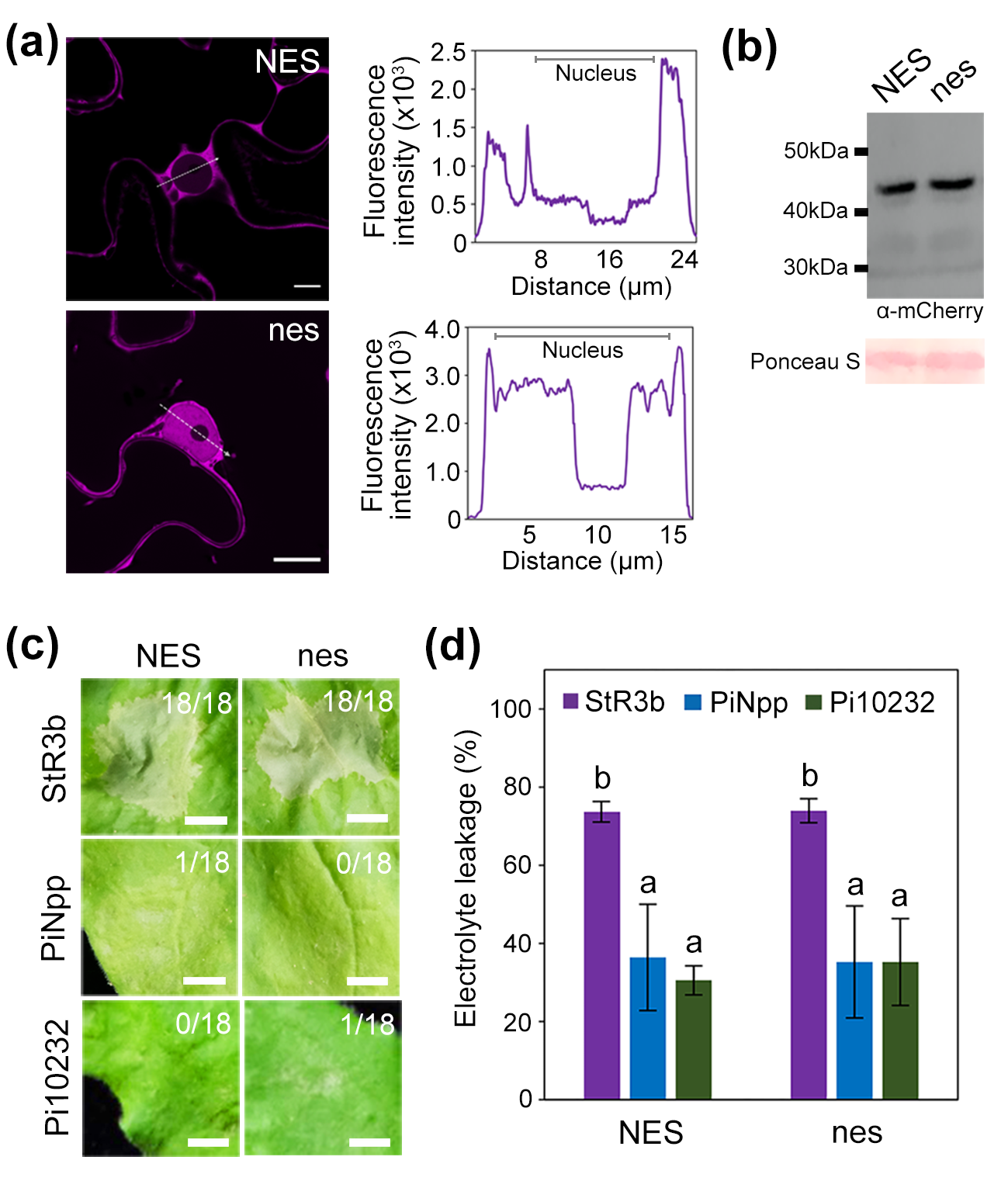

Supplement: Supplementary file 9 — Figure S9 NES‐tagged PiAvr3bRGR fails to alter StR3b‐induced hypersensitive response and PiNp‐ or Pi10232‐triggered cell death. (a) Subcellular localization of PiAvr3bRGR fused with an N‐terminal nuclear export signal (NES) or a null functional form (nes) in Nicotiana benthamiana. Images were captured at 2‐3 days postagroinfiltration (dpa). Scale bar, 10 μm. Fluorescence density across the cytoplasm and nucleus (grey bar) was measured and compared, as shown on the right panel. Three independent confocal microscopy observations (n > 18) were made, and representative images were shown. (b) Western blot analysis of expressed fusion proteins with α‐mCherry. Protein loading was indicated by Ponceau S staining. (c) N. benthamiana leaves showing cell death triggered by StR3b, PiNpp, or Pi10232 and mislocalization mutants of PiAvr3b. Photographs were captured at 3–5 dpa. The relationship between the cell death phenotype and the total number of infiltrations (n = 18) is shown. Assays were repeated at least three times with similar results. (d) Quantification of cell death by measuring electrolyte leakage in (c). Electrolyte leakage from infiltration sites was measured as a percentage of leakage from boiled samples. Kruskal‐Wallis test was used for statistical analysis with a p‐value cutoff of 0.05. Letters at the top of each bar indicate statistically significant differences. [file MPP-24-317-s008.tif]

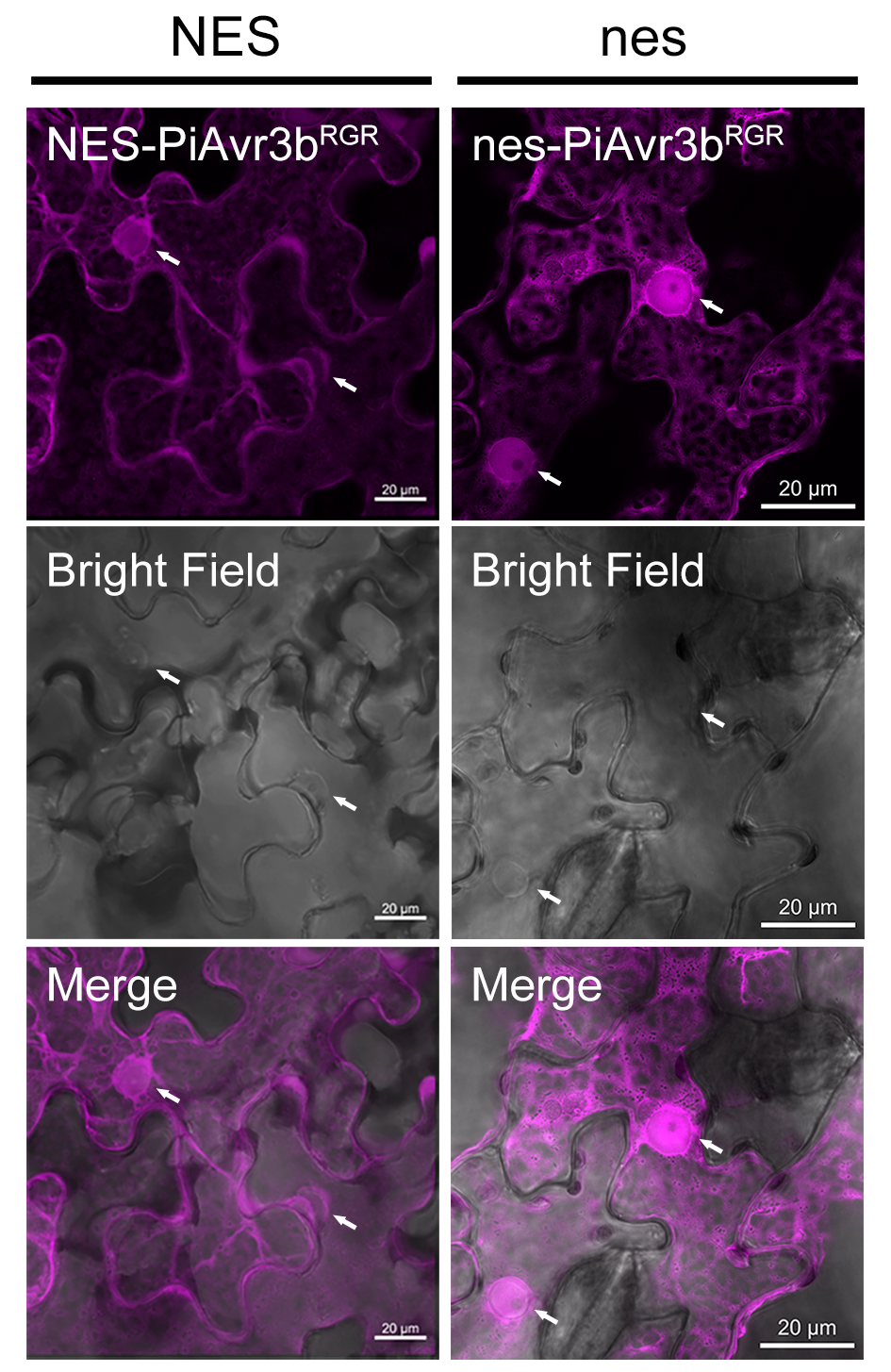

Supplement: Supplementary file 10 — Figure S10 Confocal microscopy showing Nicotiana benthamiana leaves with transient expression of NES‐ and nes‐tagged mCherry‐PiAvr3bRGR. White arrows indicate nuclei. Scale bar, 20 μm. Two independent confocal microscopy observations (n > 12) were made, and representative projection images were shown. [file MPP-24-317-s012.tif]

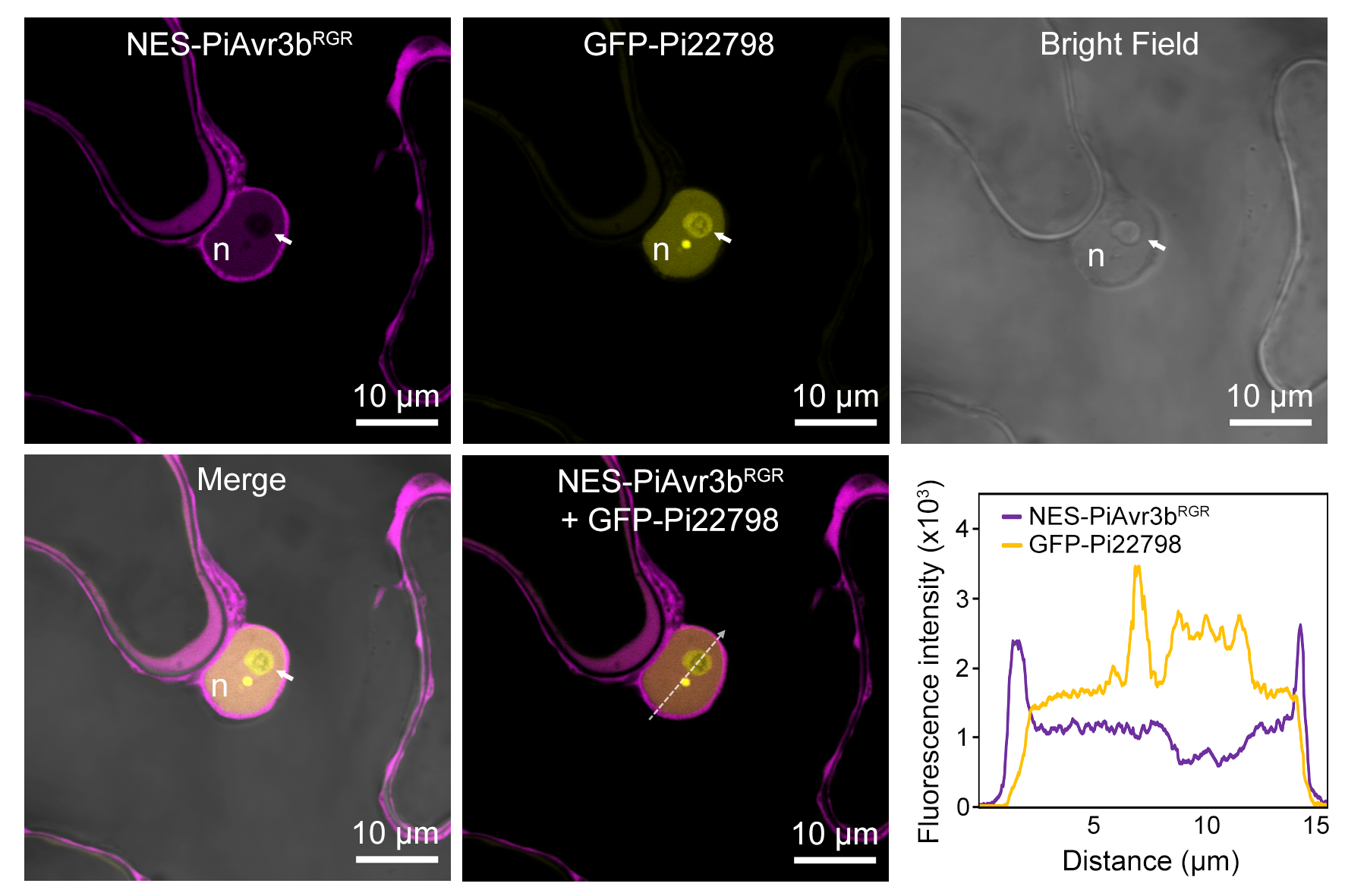

Supplement: Supplementary file 11 — Figure S11 Co‐localization of NES‐tagged mCherry‐PiAvr3bRGR and GFP‐Pi22798 in Nicotiana benthamiana leaves. n: nucleus; arrow indicates nucleolus. Scale bar, 10 μm. Two independent confocal microscopy observations (n > 18) were made, and representative images were shown. The fluorescence density across the cytoplasm and nucleus was measured and compared, as shown on the bottom right panel. [file MPP-24-317-s014.tif]
